# Chronic unilateral vestibular hypofunction: a qualitative study exploring the full spectrum of symptoms and impacts through the ICF framework

**DOI:** 10.3389/fneur.2025.1589404

**Published:** 2025-06-02

**Authors:** Mustafa Karabulut, Hamide Avci, Eda Yalçınkaya, Merel Kimman, Wolfgang Viechtbauer, Alfarghal Mohamad, Vincent Van Rompaey, Nils Guinand, Angélica Perez Fornos, Levent Özlüoğlu, Osman Nuri Özgirgin, Raymond van de Berg

**Affiliations:** ^1^Division of Vestibular Disorders, Department of Otorhinolaryngology and Head and Neck Surgery, School for Mental Health and Neuroscience, Maastricht University Medical Center, Maastricht, Netherlands; ^2^Faculty of Behavioural and Social Sciences, Department of Pedagogy and Educational Sciences, University of Groningen, Groningen, Netherlands; ^3^Department of Otolaryngology-Head and Neck Surgery, Bayındır Hospital, Ankara, Türkiye; ^4^Department of Clinical Epidemiology and Medical Technology (KEMTA), Care and Public Health Research Institute (CAPHRI), Maastricht University Medical Centre, Maastricht, Netherlands; ^5^Department of Psychiatry and Neuropsychology, Maastricht University, Maastricht, Netherlands; ^6^Department of Ear Nose Throat, King Abdul Aziz Medical City, Jeddah, Saudi Arabia; ^7^Department of Otorhinolaryngology and Head & Neck Surgery, Antwerp University Hospital, Faculty of Medicine and Health Sciences, University of Antwerp, Antwerp, Belgium; ^8^Service of Otorhinolaryngology Head and Neck Surgery, Department of Clinical Neurosciences, Geneva University Hospitals, Geneva, Switzerland; ^9^Department of Otorhinolaryngology and Head and Neck Surgery, Ankara Baskent University Hospital, Ankara, Türkiye

**Keywords:** unilateral vestibular hypofunction, unilateral vestibular loss, unilateral vestibulopathy, symptoms, international classification of functioning, disability and health (ICF), patient-reported outcome measure (PROM), quality of life

## Abstract

**Aim:**

To explore the full spectrum of symptoms and impacts associated with chronic unilateral vestibular hypofunction (UVH), and to assess whether these symptoms and impacts are fully covered by patient-reported outcome measures (PROMs) within the International Classification of Functioning, Disability and Health (ICF) framework.

**Methods:**

A qualitative study was conducted using semi-structured interviews, which were recorded, transcribed, and analyzed by two independent reviewers through a consensus approach. Data collection continued until thematic saturation was reached. Domains were then identified from interviews and PROMs (Dizziness Handicap Inventory, Hospital Anxiety and Depression Scale, EQ-5D-5L) using the ICF linking process. The analysis comprised three stages: (1), documenting the full spectrum of UVH symptoms and impacts from interviews, (2) reporting domains and constructs based on interviews, (3) comparing domains identified from interviews with those from PROMs (DHI, HADS, EQ-5D-5L, analyzed separately).

**Results:**

Fifteen patients with chronic UVH were interviewed. Reported symptoms revealed 16 physical symptoms, four cognitive symptoms, and five emotions. Key challenges included driving difficulties, darkness, sleep problems, fear of falling, and discomfort in crowded environments. Patients adapted certain behaviors, such as moving slowly, using supports, and avoiding sudden movements. Regarding the ICF framework, the most frequently reported construct was Body functions, with key domains including emotional, vestibular, and hearing-related functions. Activities and participation focused on maintaining body position and family relationships, while environmental factors highlighted the impact of light and sound. Interviews identified key domains related to vision, memory, multitasking, and activities impacting quality of life that were often overlooked by the PROMs.

**Conclusion:**

Patients with chronic UVH experience a wide spectrum of physical, cognitive, and emotional symptoms, resulting in significant limitations in daily life. The frequently used PROMs (DHI, HADS, and EQ-5D-5L) do not fully cover these symptoms and their impacts, which leave many aspects underrepresented. A tailored PROM for UVH may be needed, to better reflect the specific symptoms, behaviors and functional limitations related to chronic UVH.

## Introduction

Unilateral vestibular hypofunction (UVH) is a complex disorder in which vestibular function in one ear is partially or completely absent (1). The onset of UVH may occur suddenly or gradually, depending on the underlying etiology (2). UVH symptoms include, e.g., dizziness, unsteadiness and oscillopsia. These symptoms can occur in both static and dynamic conditions (3). When UVH occurs, a neurological process known as vestibular compensation, can (partially) mitigate these symptoms. Vestibular compensation targets both static and dynamic conditions. Static conditions refer to situations without head movements. Dynamic conditions refer to situations with head movements (4, 5). While vestibular compensation addresses both conditions, symptoms in static conditions (e.g., spontaneous nystagmus) typically resolve more quickly and completely (6, 7). However, 29–66% of patients continue to experience symptoms in dynamic conditions, often resulting in a chronic disorder (8–11).

Multiple etiologies can result in UVH, including Menière’s Disease, infection/inflammation, vestibular migraine, vascular conditions, or iatrogenic factors (2, 12, 13). This implies that patients with chronic UVH might experience symptoms related to different disorders. For example in patients with Menière’s disease, ‘vertigo attacks’ would be related to Menière’s disease, and ‘unsteadiness in between attacks’ would be related to UVH resulting from Menière’s disease (14). Previously, it was found that UVH can result in a spectrum of symptoms, beyond dizziness and unsteadiness (2, 12). Evaluation of therapeutic interventions (e.g., rehabilitation (15), the vestibular implant (16)) should therefore incorporate this spectrum of symptoms, to better estimate the effects of interventions.

Several techniques are used to collect UVH-related symptoms, in clinical setting and in research setting. These techniques include, for example, history taking (14), patient-reported outcome measures [PROMs; e.g., Dizziness Handicap Inventory (DHI) (17), Hospital Anxiety and Depression Scale (HADS) (18)], and interviews (19). Each method has its own strengths and limitations. For instance, PROMs provide valuable insights and allow for symptom quantification, but they may fail to assess less common symptoms (20). In previous studies, including a systematic review and a retrospective study, symptoms were collected through PROMs and self-reports; however, a full representation of UVH symptoms was still not achieved (2, 12). Therefore, conducting interviews with patients would be essential to more accurately define the entire range of UVH symptoms. The findings of these interviews could indicate whether existing PROMs would be sufficient to cover the relevant UVH symptoms. If not, a new PROM might be needed to evaluate effects of therapeutic interventions in chronic UVH patients.

Symptom definitions are crucial for effective communication in both clinical practice and research (21). Patients and clinicians might not always be on the same page when describing symptoms, which can create gaps in understanding the full impact of the condition (22–24). For example, patients may use terms like vertigo and dizziness interchangeably. Beyond symptom descriptions, it is equally important to examine patients’ behaviors, challenges, and coping strategies to fully understand their experiences. Here, the International Classification of Functioning, Disability, and Health (ICF) framework provides a structured framework to assess these aspects. It uses a standardized language to describe interactions between health conditions and the environment (25). Its universal language enhances inter- and multidisciplinary communication in both clinical and research settings and enables the comparison of health states across countries and disciplines. The ICF framework covers four key constructs: body functions, body structures, activities and participation, and environmental factors (26). This framework can allow for a clear evaluation of how UVH affects physical, social, and environmental functioning. Furthermore, linking PROMs data and findings from qualitative research to the ICF, helps capturing the broader impact of UVH on daily life, while providing a standardized language for evaluation (27). As a result, this approach enables clearer insights into patient needs. This supports a more accurate interpretation of UVH symptoms and their effects on quality of life.

Overall, the aim of this study was to identify the full spectrum of symptoms and their impact in patients with chronic UVH. Additionally, this study also aimed to determine whether symptoms and impacts are fully covered by PROMs within the ICF framework.

## Materials and methods

### Patients

Potential participants were first identified through the Bayındır Hospital database (Ankara, Türkiye), based on prior video head impulse test (vHIT) results. VHIT results had to comply with the following criteria: a reduced VOR gain on the affected side (<0.7), normal gain values on the healthy side (VOR gain between ≥0.8 and ≤1.2), and an asymmetry of at least 18% between the two ears. Patients who met these vHIT criteria were then contacted by phone to assess whether they experienced at least one of the following chronic symptoms: dizziness, unsteadiness, oscillopsia, or symptoms worsening with head movements. To be eligible, symptoms had to persist ≥3 months. Only patients who fulfilled both the criteria for vHIT and chronic UVH symptoms, were able to participate in the study. After providing informed consent, all participants underwent a second vHIT to confirm that they still met the diagnostic criteria. Patients who did not meet the criteria upon re-testing, or who did not report any of the required symptoms during the in-person interview, were excluded from the final sample. Other exclusion criteria included a medical history of neurological disorders (e.g., multiple sclerosis, stroke, Parkinson’s disease). Additionally, patients who were not able (e.g., mentally disabled) or willing to discuss certain topics (e.g., psychology/psychiatry, health care utilization), unable to discontinue medication for anxiety or depression (due to the vestibulo-suppressive effect), or refusing to undergo vHIT, were excluded from the study. As this study employed a qualitative approach, the goal was to explore a wide range of symptoms and their impacts, rather than their frequency. In other words, the type of symptoms was more important than how often they occurred. Therefore, patients from different ages and genders were included to reflect a diverse UVH population. All patients underwent vHIT by an experienced vestibular clinican (E.Y.), and were interviewed by the first author (M.K.).

### Vestibular testing

The vHIT was performed using the Ulmer system (Synapsis, Marseille, France). The procedure was previously described (28). In brief, the patient was seated in a chair and instructed to fixate on a target on the wall, positioned 1.5 m away. Head impulses were performed in the plane of both horizontal semicircular canals, with a velocity between 150°/s- 250°/s. The amplitudes were low (± 20°). A minimum of 10 impulses were administered in each direction. The Synapsis system calculated the VOR gain from 40 ms before to 80 ms after peak head acceleration for each impulse. In cases with covert saccades, the 80-ms window was adjusted accordingly, and stopped at time of onset of the covert saccade (29). However, the specific method used by the Synapsis system for gain calculation is not disclosed by the manufacturer. Regarding the vHIT criteria, no standardized diagnostic criteria are currently available for chronic UVH. The Bárány Society’s recommendations for acute unilateral vestibulopathy suggest a VOR gain <0.7 on the affected side and/or a side difference of >0.3 between ears. However, this side difference of 0.3 is only considered relevant when the affected side has a gain >0.7 (e.g., the affected side 0.25 and the healthy side 0.55, indicating bilateral vestibulopathy). Thus, using an asymmetry of 18% consistently corresponds to a side difference of >0.3 between the healthy and the affected side. As a result, a more conservative approach was used in our study: a gain <0.7 on the affected side and an asymmetry ≥18%.

### Research paradigm

A post-positivist approach was used to combine results of PROMs with patient interviews. An objective reality was acknowledged (vHIT demonstrated vestibular hypofunction), with a focus on how individual perspectives and social contexts shape its effects (30). The principle of ‘modified objectivity’ was used. This requires the researcher to critically reflect on, and address, their own biases and assumptions (31). Other factors were also explored, like societal norms, that might have influenced patients’ experiences (32). By incorporating these elements, this approach could offer valuable insights into how patients understand and cope with UVH (30).

### Patient interviews

Individual semi-structured interviews were conducted. Patients with chronic UVH were asked open-ended questions regarding the types of symptoms they encounter: the most frequently occurring symptoms, the symptoms that disrupt their daily lives, situations in which these symptoms are noticeable, coping strategies, and the impact of UVH on their relationships. The questions are provided in Supplementary Table S1. The interviews were conducted in person (14 patients) and online (1 patient). The protocol was reviewed by co-authors (HA, EY, ONÖ, RvdB) to optimize the interview guide. Every interview was recorded and transcribed word-for-word, without the use of any software.

Each interview was then analyzed using thematic analysis via ATLAS.ti software (33). This analysis used a “coding” process, wherein the first and second author (M.K. and H.A.) independently extracted keywords and statements from the transcriptions. These data were reviewed and discussed, which led to a consensus-based categorization into primary codes (e.g., imbalance, forgetfulness, sadness). Following this, the same authors independently developed main themes (e.g., physical symptoms, emotions, and challenging tasks) based on the primary codes, which were then labeled accordingly. Additionally, emotions were coded using Parrot’s classification of emotions, which included primary, secondary, and tertiary levels (34) (Supplementary Table S2). In case the two authors disagreed, the original data was reassessed to reach consensus. All interviews and transcriptions were conducted in Turkish, and the coding process was subsequently carried out in English. Analyses continued until saturation was reached (35–37). Mindmaps were used to visually illustrate the full spectrum of reported symptoms and impacts, created by Mindomo (9.2.4).

### Content analysis: patient-reported outcome measures

The Dizziness Handicap Inventory (DHI) (38), the Hospital Anxiety and Depression Scale (HADS) (39), and the EuroQol-5D-5L (EQ-5D-5L) (40) were used for data collection. These tools were selected since they represent different symptom domains affected by UVH, and are widely recognized and validated tools in clinical and research contexts. Specifically, the DHI is designed to assess the self-perceived impact of dizziness and/or unsteadiness on daily life across physical, functional, and emotional subdomains. The HADS examines anxiety and depression, and the EQ-5D-5L evaluates overall health status and quality of life, including pain/discomfort, mobility, self-care, usual activities, and mental well-being. These tools were also previously validated in Turkish populations (41–43).

In this study, the content analysis was conducted for each PROM. Content analysis is a systematic research method used to categorize and interpret textual data by linking it to predefined frameworks or themes (44). The purpose of including PROMs was to compare their assessed domains with those identified in patient interviews. This comparison aimed to explore potential gaps and overlaps between interviews and PROMs. In this stage, each item from PROMs was systematically evaluated and linked to its corresponding ICF constructs and domains. These terms and ICF linking procedure is described in detail below.

### ICF linking

The ICF linking procedure includes several terms: items, concepts, constructs, and domains. An *item* is a measurable element that captures specific aspects of a domain or construct, either as structured questions in PROMs or as interview quotations from participant-driven insights. For example, a PROM item might ask, “How often do you experience difficulty walking a short distance?,” while an interview quotation item might state, “I can no longer walk to the grocery store without holding onto something.” A *concept* is a variable created to represent the general meaning of an item, typically summarized in 2–3 words for clarity and categorization. For instance, the concept for the above items might be labeled as “Walking Difficulty.” A *construct* is a broad concept defining major areas of health, functioning, or context, such as Body Functions or Activities and Participation. A *domain* is a specific subcategory within a construct, grouping related aspects of health or functioning. For example, the Mobility domain, under the construct of Activities and Participation, includes tasks such as walking or climbing stairs (45).

In this study, the items from semi-structured interviews and all PROMs were organized into standardized domains and constructs within ICF. Initially, the items from these measures were obtained. Each item was examined to identify its main and additional concepts. The main concept referred to the primary focus of the item, while additional concepts included any supplementary information. These identified concepts were then linked to the most appropriate ICF domain and construct. Items not covered by the ICF were labeled as “Not covered (Nc)” and those with insufficient information to determine an ICF code were marked as “Not definable (Nd)” (27). Annotations were noted if necessary. The first and second reviewers independently conducted the linking of items from semi-structured interviews and all PROMs. Any inconsistencies in applying the ICF linking procedure between the reviewers were discussed in consensus meetings, and agreement was reached in all cases. The linking details are available in Supplementary Tables S3, S4.

### ICF linking analysis

The ICF framework includes four main constructs: Body Functions (denoted by “b”), Activities and Participation (“d”), Environmental Factors (“e”), and Body Structures (“s”). These constructs are organized into a hierarchical structure with domains ranging from the first to the fourth level, and in some cases, a fifth level. The level of each domain is identified by the number of digits following the corresponding alphabet. For instance, “b2351-Vestibular function of balance” represents a fourth-level domain, while “b235-Vestibular functions” is a third-level domain. The second-level domain is “Hearing and vestibular functions,” and the first-level domain is “Sensory functions and pain.” First- and second-level domains in the ICF are presented without numerical codes. In this study, certain items were specifically linked to fourth-level domain [e.g., “Dizziness” (b2401)] since they could be directly linked to specific ICF codes. Others were linked to the third-level domain [e.g., “Sensations associated with hearing and vestibular functions” (b240)] in case a direct code linkage was not feasible. To maintain consistency during analysis, items initially categorized at the fourth^−^level domain, were standardized to the third-level domain. Following this, second and first-level domains, along with the constructs, were created to provide a comprehensive framework for the analysis.

**Figure 1:**
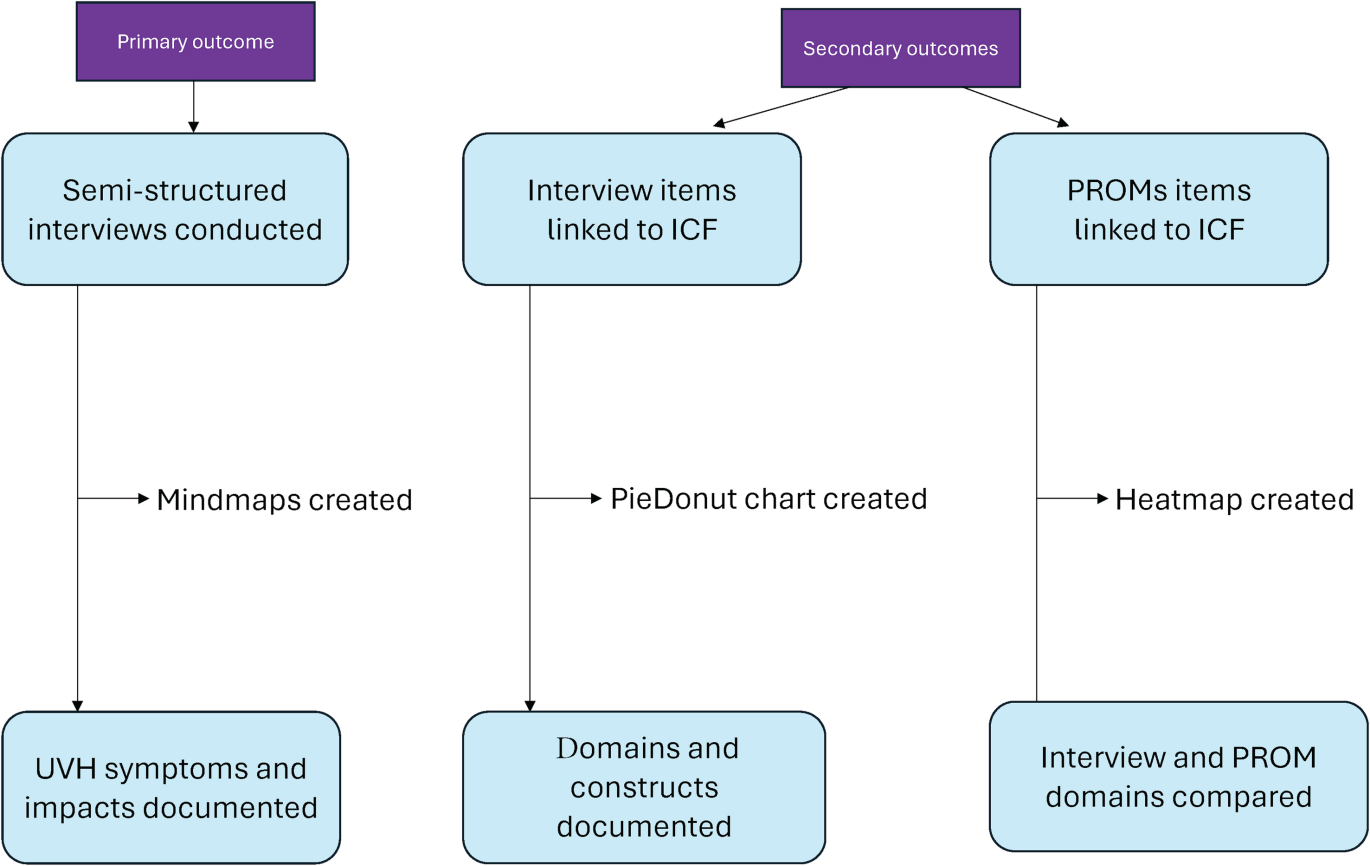
Flowchart of the study design.

Two distinct analyses were conducted. First, the frequencies of each domain and construct were analyzed using interview data and visualized through PieDonuts graph based on second-level domains. Second, domains identified from interviews were compared with those identified from PROMs (DHI, HADS, EQ-5D-5L, analyzed separately), illustrated by a heatmap. These comparisons used third-level domains to demonstrate the more detailed aspects of the data. The flowchart of the study design is shown in Figure 1.

### Ethical considerations

This study adhered to the legislation and ethical standards on human experimentation in Türkiye, as well as the principles outlined in the Declaration of Helsinki. The medical ethical committee of Bayındır Söğütözü Hospital approved this study (BTEDK-03/23 date: 02.02.2023), and written informed consent was obtained from all patients.

## Results

### Patient characteristics

Fifteen chronic UVH patients (9 women, 6 men) with a mean age of 64 years (range 38–75 years), were included for the qualitative analysis. The duration of symptoms ranged from 7 months to 24 years. The diagnoses of UVH in these patients were: Acute unilateral vestibulopathy/vestibular neuritis (*n* = 6); Menière’s disease (*n* = 5); idiopathic (*n* = 3) and vestibular schwannoma (*n* = 1). Based on vHIT results, 6 patients had a right sided UVH and 9 patients had a left sided UVH. The mean VOR gain on the pathological side was 0.33 (SD ± 0.21), while the healthy side showed a mean value of 0.86 (SD ± 0.06). The average asymmetry (%) between the two sides was 48 (SD ± 25.8). Regarding co-morbidities, more than half of the patients (53%) had hypertension (*n* = 8). Additionally, patients reported migraine headache, non-migraine headache, anxiety, or autoimmune disorders (each *n* = 3). The least reported co-morbidities were diabetes and depression (13%, *n* = 2). Table 1 presents the results of each PROM (DHI, HADS and EQ-5D-5L) administered to 15 chronic UVH patients. The DHI showed a mean score of 49.2 ± 16.5, indicating a moderate handicap, with scores ranging from mild to severe handicap (range 28–80). The physical subscale revealed the highest score (18.6 ± 5.9) out of max. 28 points. The HADS indicated borderline anxiety levels (7.5 ± 3.9) and normal depression levels (6.3 ± 4.0). The EQ-5D-5L showed a mean index value of 0.580 ± 0.19, which is lower than the Dutch age-adjusted reference value of 0.839 (60–70 years). Among the five dimensions of the EQ-5D-5L, the “anxiety/depression” dimension was most affected, with a mean score of 2.9 ± 1.0 out of 5.

**Table 1:** Results from the DHI, HADS and EQ-5D-5L obtained in 15 chronic UVH patients.

Questionnaire	Mean (SD; Median, range)
DHI	
Physical	18.6 (SD = 5.9) (20, 8–28)
Functional	16.6 (SD = 7.4) (14, 6–29)
Emotional	14.0 (SD = 7.3) (14, 2–28)
Total	49.2 (SD = 16.5) (46, 28–80)
HADS	
Anxiety	7.5 (SD = 3.9) (7, 1–16)
Depression	6.3 (SD = 4.0) (6, 1–13)
EQ-5D-5L	
VAS	62.7 (SD = 14.43) (60, 25–80)
Index value	0.580 (SD = 0.19) (0.620, 0.213–0.883)

Reference values of questionnaires: DHI: subscales: physical (max 28), functional (max 36), and emotional (max 36). Total score (range 0–100): ≤ 16 no handicap, 16–34 mild handicap, 36–52 moderate handicap, ≥ 54 severe handicap. HADS: 0–7 normal, 8–10 borderline, 11–21 abnormal.EQ-5D-5L: VAS = visual analog scale (0—100%), Mean index value score for age. 50–60 years 0.857 and 60–70 years: 0.839. SD = Standard deviation.

### Patient interviews

After 15 interviews, no new information was obtained and transcription was discontinued. The average duration of the interviews was 31 min (range 21–52 min) and resulted on average in 2739 words (range 2,166–4,413 words). Through thematic analysis of participants’ responses, four main categories were identified: symptoms (physical, cognitive, emotions), challenging tasks, coping strategies and behavior. While the interview questions broadly reflected these areas, the final categorization resulted from the individual interview data. The associated frequencies of occurrence can be found in Supplementary Table S5.

### Physical symptoms

Chronic dizziness and unsteadiness were among the most frequently reported symptoms, affecting the majority of patients. Many reported unsteadiness while walking, when bending over, and with fast body movements. Moreover, some noted that darkness exacerbated their symptoms, requiring to turn on lights while walking or standing. To maintain stability, patients indicated the need of support or a reference point, such as holding onto an object.

Another commonly described symptom was visually-induced dizziness, also known as ‘the supermarket effect’, often triggered in environments with complex visual stimuli. More than half of patients described difficulties when looking at fast-moving objects, car headlights, certain patterns, colors, or objects that move like flowing water. Many also struggled with the inability to perform fast head movements during activities such as walking through pedestrian crossings or looking in mirrors while driving.

Some patients reported autonomic complaints/orthostatic dizziness, experiencing short-term dizziness when making sudden movements from sitting to standing or lying down to standing, requiring them to wait for a moment. Tiredness was also reported by several patients in a way that fatigue occurred in the absence of physical or mental effort, with increased frequency and intensity since UVH onset.

**Figure 2:**
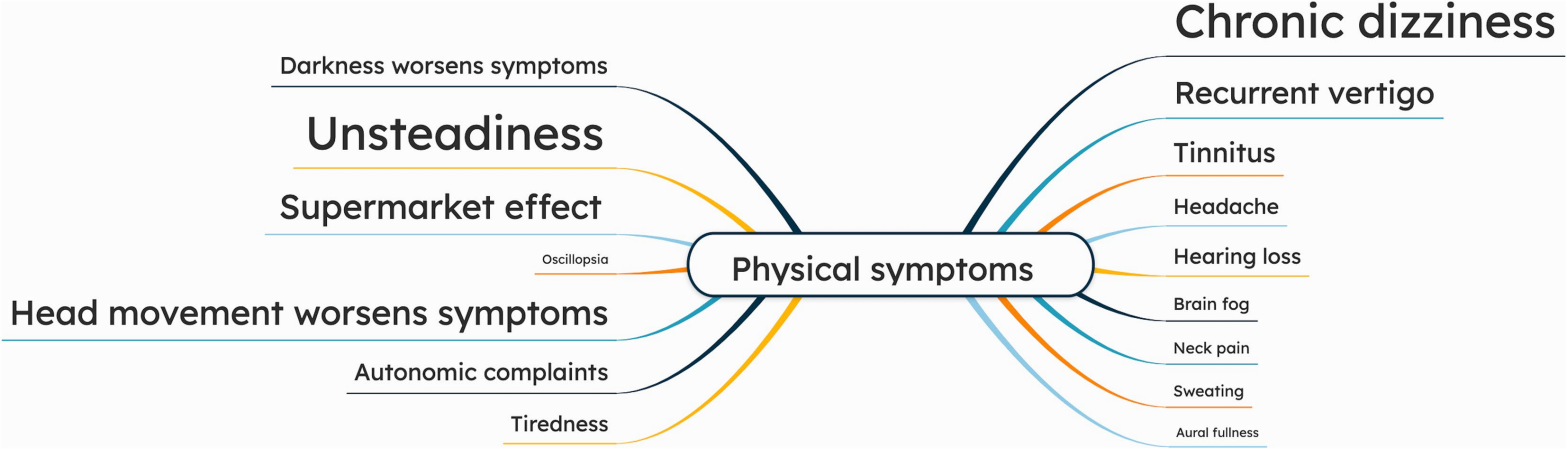
Mind map of physical symptoms, as reported by chronic UVH patients (*n* = 15) during semi-structured interviews. The larger the font size of a specific symptom, the more often this symptom was addressed during the interviews.

Only few patients mentioned oscillopsia, who described a moving horizon or environment during movements, and difficulty reading signs while walking. Additionally, participants reported other symptoms such as recurrent vertigo, tinnitus, headache, hearing loss, brain fog, neck pain, sweating, and aural fullness. Figure 2 presents a mind map illustrating the physical symptoms. The patient quotations related to cognitive symptoms are presented in Supplementary Table S3.

### Cognitive symptoms

**Figure 3:**
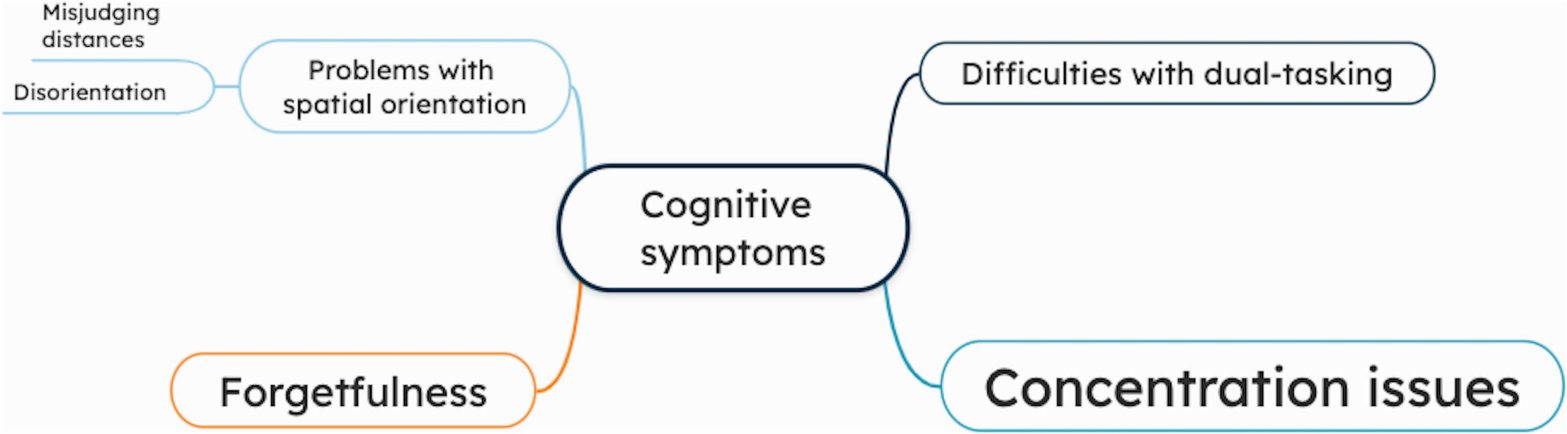
Mind map of cognitive symptoms, as reported by chronic UVH patients (*n* = 15) during semi-structured interviews. The larger the font size of a specific symptom, the more often this symptom was addressed during the interviews.

The majority of patients reported difficulties with concentration, describing struggles with following long conversations, the need to re-read passages for comprehension, and difficulty to repeatedly perform familiar tasks. More than half of patients also noted increased forgetfulness, which was reflected in challenges with recalling familiar information, confusion during routine activities, and forgetting daily tasks. Several patients experienced difficulties with dual tasking, such as walking while reading on their smartphone, or generally trying to do two things at the same time. The least reported cognitive symptom was spatial orientation, which was further categorized into two types: disorientation and misjudging distances. Misjudging distances was illustrated by examples such as bumping into objects or struggling with spatial awareness while driving. Figure 3 presents a mind map illustrating the cognitive symptoms. The patient quotations related to cognitive symptoms are presented in Supplementary Table S3.

### Emotions

Parrot’s classification of emotions was used to categorize the emotions into primary, secondary, and tertiary emotions. All patients reported negative emotions, and some mentioned positive emotional experiences.

Sadness was often described. It was categorized into disappointment, neglect, sadness, and suffering. Disappointment reflected unmet expectations (e.g., “I did not expect to have this illness” [UVH-6]). Neglect included embarrassment, insecurity, and isolation (e.g., “I feel concerned if others notice when I stumble” [UVH-3], “This condition (UVH) makes me feel trapped” [UVH-7]). Sadness encompassed depression, despair, sadness, and woe (e.g., “Constant dizziness makes me feel I’ve lost control” [UVH-8], “I asked the doctor if I could drive a car again” [UVH-11]). Lastly, suffering was characterized by an overwhelming emotion of suffering (“It feels like my mind is constantly heavy, and I cannot find any relief.”[UVH-10]).

Fear was also frequently mentioned, categorized into nervousness and horror. Nervousness included anxiety, distress, and worry with patients fearing daily activities (e.g., “I’m afraid of going out alone, feel anxious about facing the same challenges” [UVH-1], and “I’m worried about falling or hurting myself” [UVH-7]). Horror involved fear and panic, such as, “I try to avoid darkness; it terrifies me” [UVH-5], “I suddenly feel overwhelmed, like my heart is racing and I cannot catch my breath.” [UVH-2].

More than half of patients expressed anger, primarily in the form of frustration, irritation and exasperation. Frustration included statements like “I can react sharply to the smallest things” [UVH-1]. Irritation was reflected in a comment such as “Restricted movements irritate me” [UVH-10].

**Figure 4:**
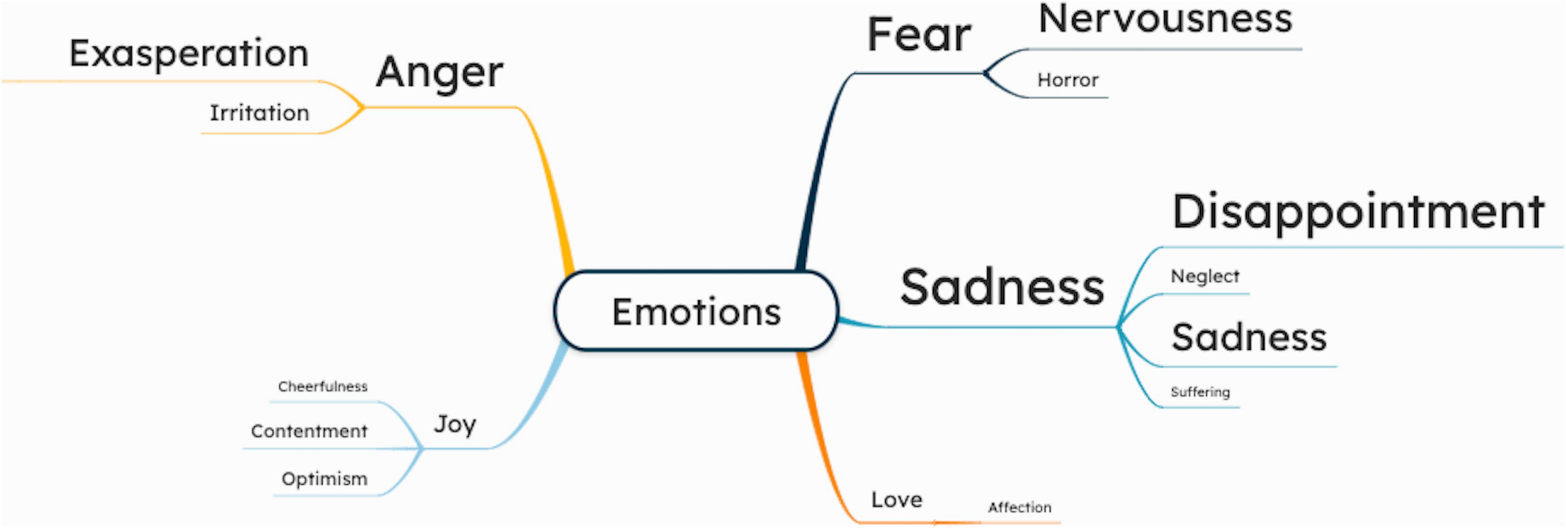
Mind map of cognitive symptoms, as reported by chronic UVH patients (*n* = 15) during semi-structured interviews. The larger the font size of a specific symptom, the more often this symptom was addressed during the interviews.

Several patients also reported positive emotions, including joy and love. Joy, expressed as cheerfulness, contentment, and optimism, included statements as “At least I’m happy that this disease is gradually getting better.” [UVH-8], and “I have learned to appreciate life and be content with what I have” [UVH-9]. Love was reflected in the form of affection such as “I care for my family; their company brings me joy” [UVH-4] and “Support from my close family keeps me going” [UVH-6]. Figure 4 presents a mind map illustrating the emotions. The patient quotations related to emotions are presented in Supplementary Table S3.

### Challenging tasks, coping strategies, and behavior

Driving was challenging for several patients due to various factors: visually-induced dizziness [UVH-12], difficulty turning their heads [UVH 5, 7], worsening symptoms on uneven ground, sharp turns, or sudden head movements triggered by the vehicle’s swaying motion [UVH-2, 6, 8, 15]. Some patients experienced sleeping problems, and several patients described a fear of falling particularly in darkness, on stairs, or when dual tasking. Discomfort in crowded situations was also noted, with patients finding it difficult to navigate busy environments. A few patients struggled with reading, particularly with subtitles on television.

Patients adopted various coping strategies to manage symptoms. Many emphasized moving slowly and cautiously, such as waiting before walking, or holding onto railings when using stairs (e.g., “I now think that I will do everything without rushing, taking slow steps” [UVH-2]). Patients reported to avoid sudden movements, overexertion, and crowded environments: “I try to protect myself as much as possible, avoiding rushing, overexertion, and crowded environments” [UVH-2]. At night, patients often used walls for support when moving: “At night, I support myself by holding onto walls” [UVH-10]. Others described modifying tasks to prevent discomfort, such as avoiding certain head movements: “I never lie on my left side, and when I need to look to the left, I turn my whole body” [UVH-15].

**Figure 5:**
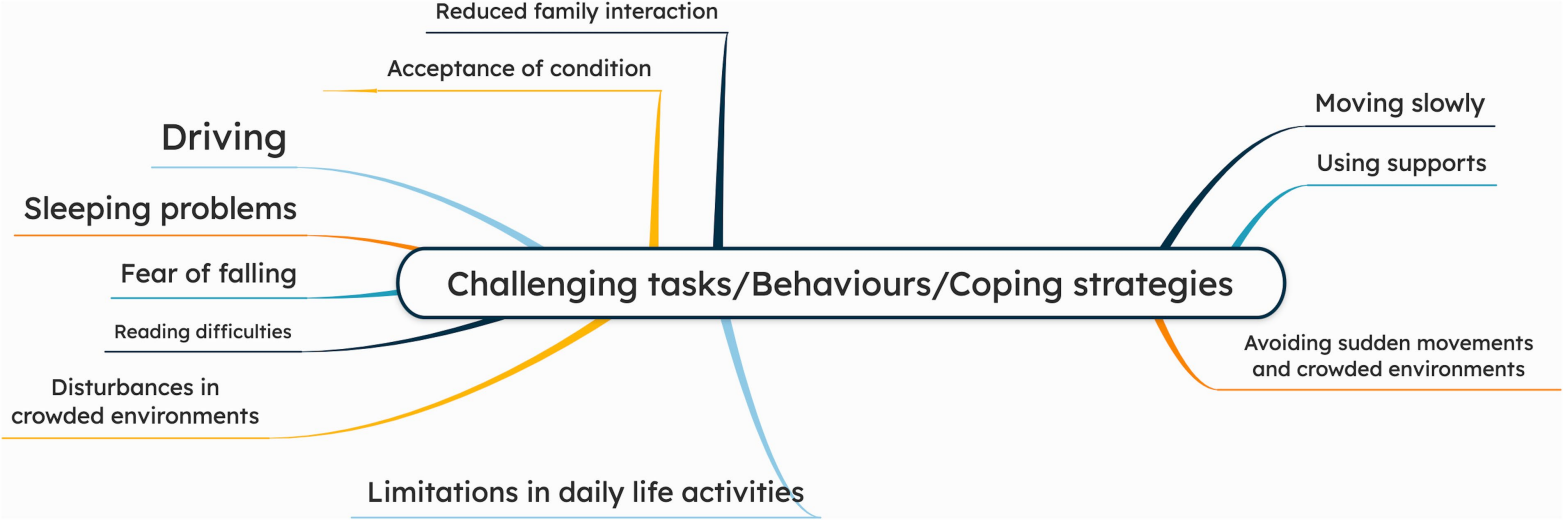
Mind map of challenging tasks, behaviors and coping strategies, as reported by chronic UVH patients (*n* = 15) during semi-structured interviews. The larger the font size of a specific symptom, the more often this symptom was addressed during the interviews.

Regarding behavior, several patients expressed acceptance of their condition, linking challenges to age or medical history, with comments such as “I accept that as I get older, certain difficulties will arise” [UVH-8], or “I have learned to live with it” [UVH-11]. A few noted reduced family interaction, such as “Even the time I spend with my child has changed” [UVH-2]. Limitations in daily life were also reported, affecting work, travel, and social activities. One described “This illness made life feel empty, like being imprisoned.” [UVH-7], while another mentioned reduced socializing: “I used to meet neighbors often, but this condition has affected me” [UVH-12]. Figure 5 presents a mind map illustrating the challenges, coping strategies and behaviors. The patient quotations related to challenges, behaviors and coping strategies are presented in Supplementary Table S3.

Table 2 shows the overview of key themes identified from semi-structured interviews with 15 chronic UVH patients.

**Table 2:** Semi-structured interview results of the 15 chronic UVH patients.

Patients	Semi-structured interview					
	Physical symptoms	Cognitive symptoms	Emotions	Challenging tasks	Coping strategies	Behaviors
UVH-1	Chronic dizziness Unsteadiness Tiredness	Forgetfulness	Sadness (Sadness) Anger (Exasperation) Fear (Nervousness)		Avoid going outside alone Limit the duration of standing upright	
UVH-2	Chronic dizziness Unsteadiness Supermarket effect Head movement worsens symptoms Oscillopsia	Concentration issues Forgetfulness	Sadness (Sadness, disappointment, neglect) Fear (Nervousness, horror)	Driving Discomfort in crowded situations Reading difficulty	Move slowly Avoid crowded environments	Reduced family interaction Limitations in daily life activities
UVH-3	Chronic dizziness Supermarket effect Head movement worsens symptoms Autonomic complaints Tiredness	Concentration issues Difficulties in dual-tasks Disorientation	Sadness (Sadness, disappointment, neglect) Anger (Exasperation, Irritation) Fear (Nervousness)		Move slowly	Reduced family interaction
UVH-4	Unsteadiness Supermarket effect Head movement worsens symptoms Darkness worsens symptoms Autonomic complaints Tiredness	Concentration issues Difficulties in dual-tasks	Sadness (Sadness, disappointment) Anger (Exasperation) Joy (Optimism) Love (Affection)	Reading difficulty	Move slowly and cautiously, pausing before walking Use railings for support when using stairs	
UVH-5	Chronic dizziness Unsteadiness Supermarket effect Head movement worsens symptoms Darkness worsens symptoms	Forgetfulness, Difficulties in dual-tasks Disorientation Misjudging distance	Anger (Exasperation) Fear (Nervousness, horror)	Driving Sleep problem	Avoid dark environments Seek support by holding onto nearby objects when needed	Limitations in daily life activities
UVH-6	Chronic dizziness Tiredness	Concentration issues	Sadness (Disappointment) Anger (Exasperation) Love (Affection)	Driving Sleep problem		
UVH-7	Chronic dizziness Unsteadiness Supermarket effect Head movement worsens symptoms Autonomic complaints Tiredness	Concentration issues Misjudging distance	Sadness (Sadness, disappointment) Fear (Nervousness) Joy (Optimism)	Driving Sleep problem	Move slowly Use railings for support when using stairs	Limitations in daily life activities
UVH-8	Chronic dizziness Unsteadiness Supermarket effect Head movement worsens symptoms Darkness worsens symptoms	Concentration issues Difficulties in dual-tasks	Anger (Exasperation) Joy (Cheerfulness, Contentment)	Driving Fear of falling Discomfort in crowded situations	Avoid crowded environments	Acceptance of condition
UVH-9	Chronic dizziness Supermarket effect Darkness worsens symptoms	Forgetfulness	Sadness (Disappointment) Fear (Nervousness) Joy (Contentment)	Sleep problem Fear of falling	Move slowly and cautiously, pausing before walking Avoid sudden movements	
UVH-10	Chronic dizziness Unsteadiness Head movement worsens symptoms	Concentration issues Forgetfulness	Sadness (Suffering, disappointment) Anger (Exasperation, Irritation) Fear (Nervousness) Love (Affection)	Sleep problem Fear of falling	Use railings for support when using stairs Avoid sudden head movements	Limitations in daily life activities
UVH-11	Unsteadiness Head movement worsens symptoms Darkness worsens symptoms		Sadness (Sadness)	Sleep problem Discomfort in crowded situations	Move slowly and cautiously Use railings for support when using stairs	Acceptance of condition
UVH-12	Chronic dizziness Unsteadiness Supermarket effect Head movement worsens symptoms Autonomic complaints	Concentration issues	Sadness (Sadness, disappointment)	Driving	Use railings for support when using stairs Avoid from light	Limitations in daily life activities
UVH-13	Unsteadiness Supermarket effect		Sadness (Disappointment) Fear (Nervousness)		Limit the duration of standing upright	
UVH-14	Chronic dizziness Unsteadiness Supermarket effect Head movement worsens symptoms Darkness worsens symptoms Autonomic complaints Oscillopsia	Concentration issues Forgetfulness	Fear (Nervousness)	Fear of falling Discomfort in crowded situations Reading difficulty	Move slowly and cautiously Use railings for support when using stairs Avoid sudden movements	Reduced family interaction
UVH-15	Chronic dizziness Unsteadiness Supermarket effect Head movement worsens symptoms Autonomic complaints	Concentration issues	Anger (Irritation)	Driving Fear of falling	Avoid certain head movements	Acceptance of condition

### ICF linking

**Figure 6:**
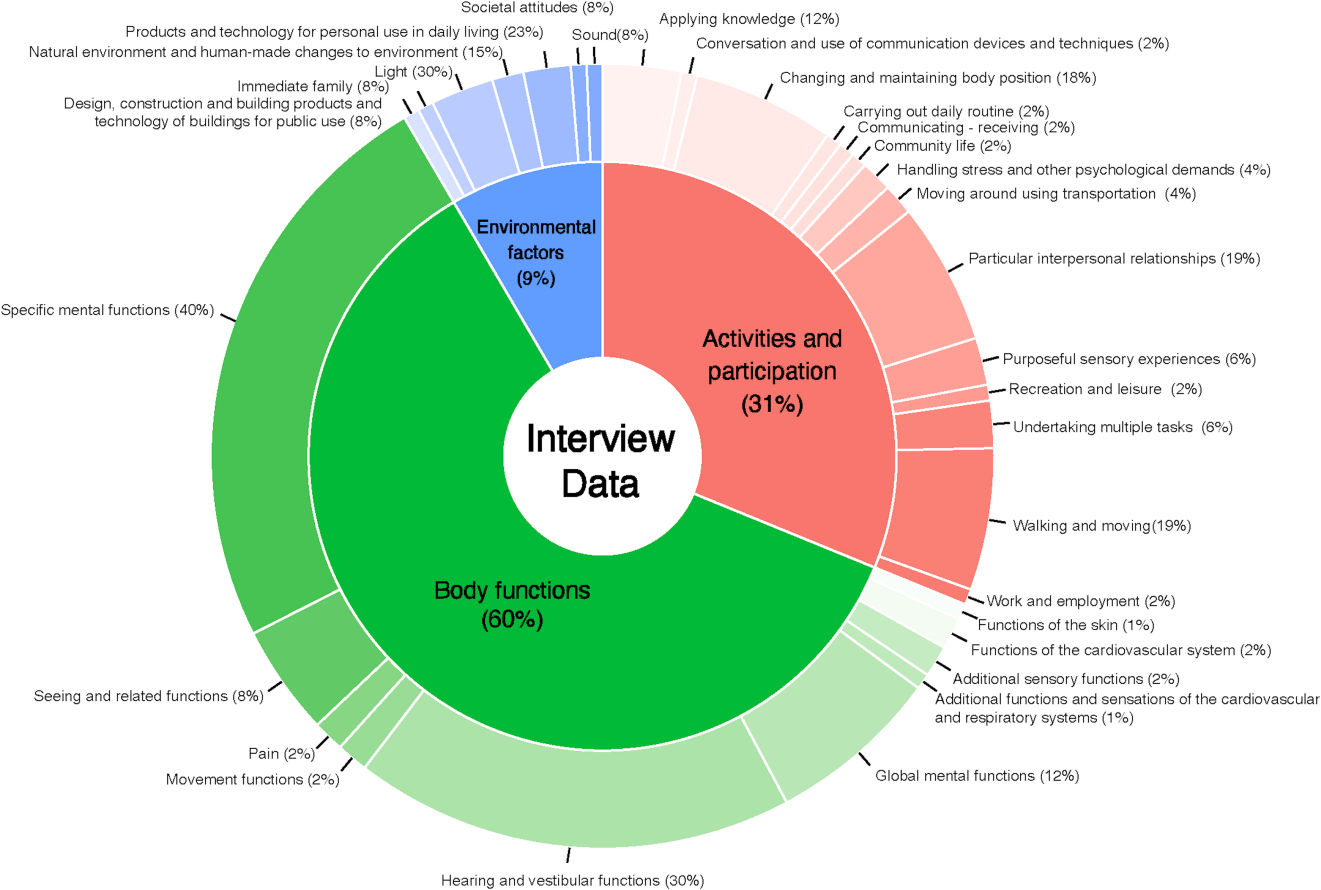
The frequencies of each construct and domain identified from semi-structured interviews.

Figure 6 illustrates the frequencies of each construct and domain identified from interviews. Body functions was the most frequently reported construct (60%). Within this construct, the most commonly reported domains were “Specific mental functions” and “Hearing and vestibular functions,” with specific items “b152-Emotional functions,” “b235- Vestibular functions,” “b240 Sensations associated with hearing and vestibular functions.” Patients reported items covering 5 out of 8 first-level domains in the ICF framework, excluding “Voice and speech functions,” “Functions of the digestive, metabolic and endocrine systems” and “Genitourinary and reproductive functions.”

Activities and participation was the secondly most reported construct (31%). The most frequently noted domains were “Changing and maintaining body position” and “Particular interpersonal relationships,” with specific items such as “d4105-Bending,” “d415-Maintaining body position,” and “d760-Family relationships.” Patients mentioned items covering 7 of the 9 first-level domains in the ICF framework, excluding “Self-care” and “Domestic life.”

Environmental factors was the least reported construct (9%). The most commonly reported domain was “Natural environment and human-made changes to environment,” including specific items such as “e240- Light,” “e250-Sound.” Patients referred to items covering 4 of the 5 first-level domains in the ICF framework, excluding “Services, systems and policies.” No items related to the body structure construct were identified in the interviews.

**Figure 7:**
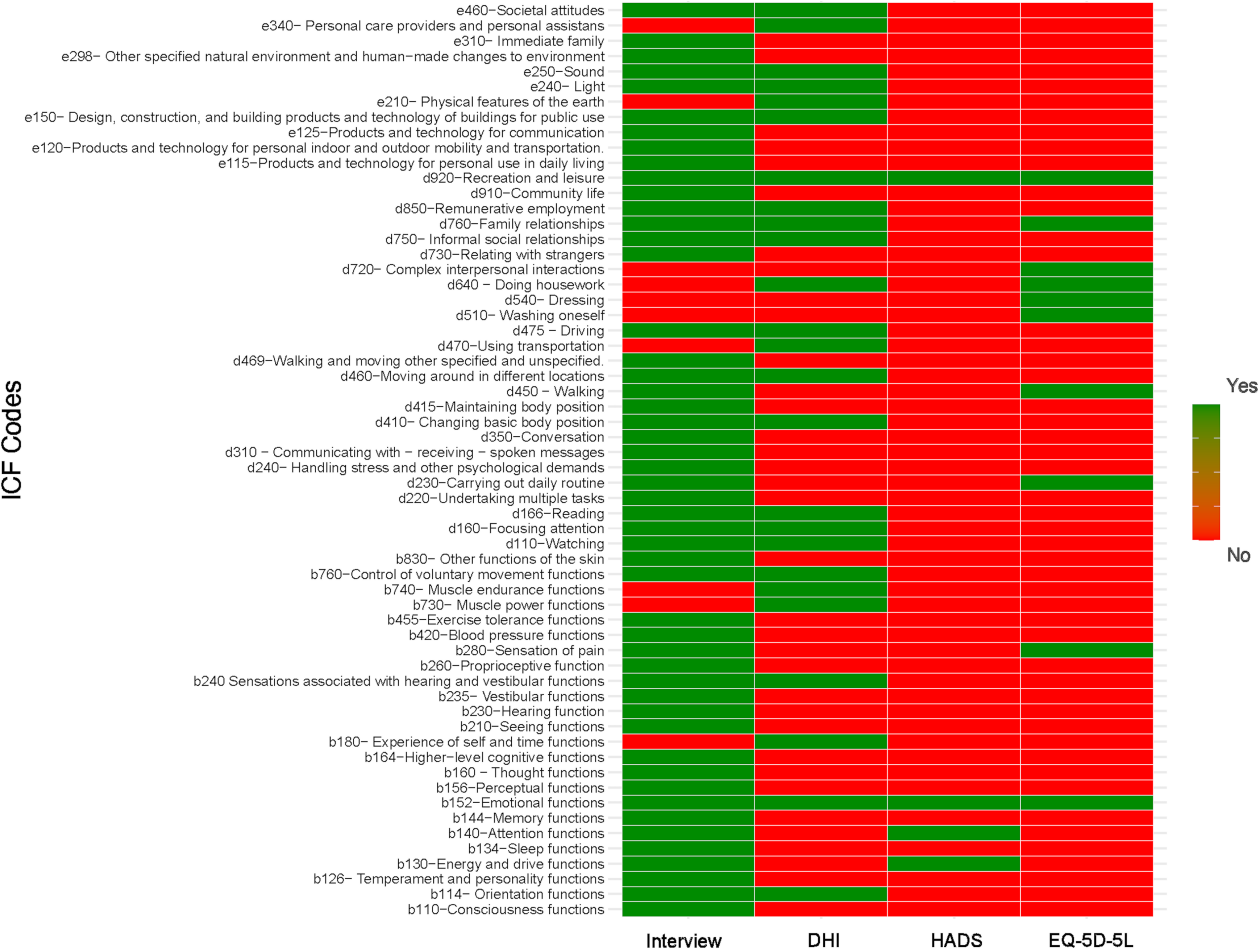
Comparative analysis of domains identified through semi-structured patient interviews versus those covered by the DHI, HADS, and EQ-5D-5L instruments.

Figure 7 compares domains identified in the patient interviews, with domains covered by the DHI, HADS, and EQ-5D-5L. The DHI does not include critical cognitive domains, such as memory and thought functions, and activities like undertaking multiple tasks. Physical limitations, including seeing function, blood pressure functions, and maintaining body position, were also absent. The HADS, which focuses on emotional aspects, does not assess physical symptoms, which limits its ability to fully represent the multidimensional impact of UVH. The EQ-5D-5L, despite providing a general overview of quality of life, does not cover key cognitive and physical domains, such as proprioception and vestibular-related challenges, making it less comprehensive in capturing the full impact of UVH on patients. The interviews, however, revealed important domains not addressed in the PROMs, including consciousness, proprioceptive, sleep, blood pressure, exercise tolerance, and hearing functions, along with community life.

## Discussion

This qualitative study demonstrated the diverse symptoms of chronic UVH, across physical, cognitive, and emotional domains. To manage symptoms, patients adopted strategies like moving slowly, using support like walls or railings, and avoiding sudden movements. However, despite these strategies, significant functional limitations in daily life, social interactions, and family relationships were found. The frequently used PROMs (DHI, HADS, and EQ-5D-5L) did not fully cover these symptoms and their impacts. This left many domains underrepresented such as vision, memory, multitasking, and daily activities impacting quality of life.

### Symptoms

Chronic dizziness and unsteadiness were among the most frequently reported symptoms in this study, which corresponds with previous research (2, 12, 46, 47). More than half of the interviewed patients reported that symptoms worsened with head movements or visual triggers. These findings may be influenced by selection bias, as patients with severe symptoms are more likely to seek medical care and participate in research. Only a few patients experienced oscillopsia, which is also congruent with previous studies (2, 3, 12, 48). Input from the remaining vestibular organ, and central vestibular compensation mechanisms, may support gaze stabilization and dynamic visual acuity but can still fail in dynamic conditions. Symptoms like recurrent vertigo and hearing loss may be linked to the underlying cause of UVH rather than UVH itself (e.g., Menière’s disease) (2, 49). Furthermore, problems with spatial orientation issues may be partially linked to hippocampal atrophy resulting from UVH (50, 51). Overall, this qualitative study supports the findings of previous studies. It prospectively confirmed that the spectrum of UVH symptoms extends beyond chronic dizziness and unsteadiness.

Some reported symptoms, for example those related to cognition and emotions, may not be exclusively due to UVH. These could be linked to other chronic conditions such as migraine, diabetes, and anxiety disorders. However, it is well established that vestibular disorders and anxiety/depression symptoms often co-exist and can mutually influence one another (52). Therefore, such symptoms were considered as part of the chronic UVH-related symptoms. Chronic conditions can heighten psychological distress and increase the need for psychosocial support. This may exacerbate negative emotional states and cognitive difficulties, such as impaired concentration and forgetfulness (53, 54). Additionally, UVH alters connectivity in cortical and subcortical brain structures, which can affect bodily self-awareness, emotional regulation, and cognitive processes (55, 56). Therefore, these symptoms likely have a multifactorial origin, reflecting both the direct effects of UVH and the broader impact of other chronic conditions.

### Challenges, coping strategies and behaviors

Patients identified several triggers that worsened their symptoms, such as overexertion, stress, and activities like bending or standing. Driving, exposure to complex visual stimuli, and crowded environments were particularly challenging, often resulting in fear of falling, heightened caution, and avoidance behaviors (57, 58). It should be noted that some triggers (e.g., exposure to complex visual stimuli and crowded environments) can also be present in other disorders than UVH, like PPPD (59). Therefore, these findings might not exclusively be related to UVH. Patients also adopted coping strategies like slowing down movements, using supports like walls or railings, and avoiding sudden movements. While these strategies may provide short-term relief, they can also reinforce maladaptive behaviors linked to PPPD, such as over-reliance on caution and hypervigilance (60). Furthermore, the significant social and emotional impacts emphasize the multifactorial nature of UVH, with common reports of frustration, isolation, and changes in family dynamics, which reflect the burden of persistent symptoms on daily life. These findings highlight the need to address both the vestibular hypofunction and the psychosocial adaptations to prevent the maladaptive behaviors and improve long-term outcomes (61). Integrating targeted interventions that consider the potential overlap with PPPD could support both functional recovery and emotional well-being (62).

### Comparison between interview and content analysis of PROMs

The interviews in this study provided detailed information, as these allowed for follow-up questions to clarify responses, reducing ambiguity caused by fixed questionnaire formats. It was demonstrated that the interviews identified several key domains which were not reflected by the DHI, HADS, and EQ-5D-5L. These domains included vision, hearing, memory, sleep, consciousness, proprioceptive, blood pressure, and multitasking.

The DHI, HADS, and EQ-5D-5L were selected for their reliability and widespread use in assessing chronic UVH. The findings of this study imply that these PROMs not fully represent the wide spectrum of symptoms experienced by UVH patients. In addition to these PROMs, a large number of other PROMs (*n* = 48) were developed for patients with vestibular symptoms (63). However, the majority of these PROMS were designed for a heterogeneous patient population, and focus on single domains such as dizziness or emotional distress (63). Furthermore, a previous qualitative study in vestibular patients found that around one-quarter of interview items raised by patients, were not reflected by existing PROMs (64). Given these limitations, it can be concluded that current PROMs not fully reflect UVH-related symptoms and their impact on daily life. Therefore, it might be preferred to develop a tailored PROM specifically designed for UVH, which takes all the relevant symptoms, behaviors and functional limitations into account (65–67).

### The role of the ICF framework

The ICF framework was not used to identify symptoms or functional limitations, but rather to systematically classify and organize them. Since the interviews provided a wide range of patient experiences, the ICF served as a structured model to categorize these findings into well-defined domains and constructs. This helped to ensure that the results were presented in a way that allows for comparison with existing health models and other conditions. In line with this, previous studies used the ICF to identify relevant domains of functioning, to develop standardized Core Sets for dizziness and balance disorders, and to explore the influence of environmental triggers on symptom severity (68–70). These efforts demonstrate the value of using a standardized recognized classification system to understand the broader impact of vestibular disorders on daily life. Following previous studies, this study applies the ICF framework to both interview data and PROMs in chronic UVH, which offered an integrated perspective on how physical, emotional, and cognitive challenges can be categorized and communicated within a standardized health model. Additionally, using the ICF framework makes it easier to communicate the impact of UVH to healthcare professionals, researchers, and policymakers by placing patient experiences within a standardized classification system. This system supports cross-study and cross-population comparisons, which can therefore aid in the identification of both common patterns and unique challenges.

This qualitative study, along with a previous systematic review and retrospective study on chronic UVH (2, 12), identified symptoms, daily life impact, and functional limitations that closely aligned with those reported in patients with bilateral vestibulopathy (BVP) (71–73). Both disorders share similar symptoms such as unsteadiness, visually-induced dizziness, oscillopsia, cognitive complaints, autonomic dysfunction, tiredness, and spatial disorientation, although their severity and impact may vary. To address these challenges in BVP, the Bilateral Vestibulopathy Questionnaire (BVQ) was developed as a PROM to evaluate the full spectrum of symptoms and their impact on daily life (74, 75). Given the substantial overlap in symptomatology and functional consequences between chronic UVH and BVP, the BVQ is currently being evaluated for validity in patients with chronic UVH.

### Limitations

Several limitations were identified in this study. First, there might be selection bias because patients were chosen using purposive sampling. This means that those who agreed to participate could have different symptoms or psychological traits compared to those who did not participate. Secondly, even though the protocol was checked by three different co-authors, there was no pilot study to test the interview questions beforehand, which could imply that important aspects of patients’ experiences may have been missed. Thirdly, no standardized diagnostic criteria for chronic UVH are currently available. Diagnosis of UVH was therefore based on horizontal vHIT in this study, which is consistent with the Bárány Society’s criteria for bilateral vestibulopathy. Horizontal vHIT was also selected due to its higher specificity, reduced susceptibility to artifacts compared to vertical vHIT or the caloric test, and its relevance to natural head movement frequencies (76, 77). While this approach may have limited sensitivity, it minimized false positives and aligned with available standards. Lastly, this study was limited to a Turkish population, which may affect its relevance to other cultures due to differences in symptom perception.

## Conclusion

Patients with chronic UVH experience a wide spectrum of physical, cognitive, and emotional symptoms, resulting in significant limitations in daily life. The frequently used PROMs (DHI, HADS, and EQ-5D-5L) do not fully cover these symptoms and their impacts, which leaves many aspects underrepresented. A tailored PROM for UVH may be needed, to better reflect the specific symptoms, behaviors and functional limitations related to chronic UVH.

## Data Availability

The original contributions presented in the study are included in the article/Supplementary material, further inquiries can be directed to the corresponding author.
