# Supplementary material for: Chronic unilateral vestibular hypofunction: a qualitative study exploring the full spectrum of symptoms and impacts through the ICF framework
Source: Front Neurol. 2025 Jun 2;16:1589404. doi: 10.3389/fneur.2025.1589404 (PMC12171127; doi:10.3389/fneur.2025.1589404)
Supplement: Supplementary file 1 [file Table_1.DOCX]

**Table S1. Interview questions**

| 1. Could you share your experiences related to your condition?  - How/When did it happen? - Was it sudden/gradual? - What symptoms did you experience? - What do you believe are the causes? |
| --- |
| 1. How long have you had your complaints/symptoms? |
| 1. What complaints/symptoms related to your balance system do you currently experience?  - Physical/Cognitive/Emotional |
| 1. What complaints/symptoms do you experience most frequently? |
| 1. Which complaints/symptoms interfere most with your daily life? |
| 1. When or in what situations do your complaints/symptoms worsen or appear? |
| 1. How do you cope with these symptoms?  - How do you feel about managing this condition? How has it impacted you? |
| 1. How do these complaints/symptoms affect your relationships with your partner/family/friends/work colleagues? |
| 1. What treatment processes have you undergone (medical treatment, vestibular rehabilitation, surgical procedures)?  - How long was each treatment? - What was your experience? |
| 1. Do you have any other additional health conditions? |

**Table S2.** Parrot’s Classification of Emotions (2001)

| Primary emotion | Secondary emotion | Tertiary emotion |
| --- | --- | --- |
| Love | Affection | Adoration, affection, love, fondness, liking, attraction, caring, tenderness, compassion, sentimentality |
|  | Lust | Arousal, desire, lust, passion, infatuation |
|  | Longing | Longing |
| Joy | Cheerfulness | Amusement, bliss, cheerfulness, gaiety, glee, jolliness, joviality, joy, delight, enjoyment, gladness, happiness, jubilation, elation, satisfaction, ecstasy, euphoria |
|  | Zest | Enthusiasm, zeal, zest, excitement, thrill, exhilaration |
|  | Contentment | Contentment, pleasure |
|  | Pride | Pride, triumph |
|  | Optimisim | Eagerness, hope, optimism |
|  | Enthrallment | Enthrallment, rapture |
|  | Relief | Relief |
| Anger | Irritation | Aggravation, irritation, agitation, annoyance, grouchiness, grumpiness |
|  | Exasperation | Exasperation, frustration |
|  | Rage | Anger, rage, outrage, fury, wrath, hostility, ferocity, bitterness, hate, loathing, scorn, spite, vengefulness, dislike, resentment |
|  | Disgust | Disgust, revulsion, contempt |
|  | Envy | Envy, jealousy |
|  | Torment | Torment |
| Sadness | Suffering | Agony, suffering, hurt, anguish |
|  | Sadness | Depression, despair, hopelessness, gloom, glumness, sadness, unhappiness, grief, sorrow, woe, misery, melancholy |
|  | Disappointment | Dismay, disappointment, displeasure |
|  | Shame | Guilt, shame, regret, remorse |
|  | Neglect | Alienation, isolation, neglect, loneliness, rejection, homesickness, defeat, dejection, insecurity, embarrassment, humiliation, insult |
|  | Symphaty | Pity, sympathy |
| Fear | Horror | Alarm, shock, fear, fright, horror, terror, panic, hysteria, mortification |
|  | Nervousness | Anxiety, nervousness, tenseness, uneasiness, apprehension, worry, distress, dread |

**Table S3.** The extracted data and linking details from semi-structured interviews

| **Name of symptoms** | **Verbatim health information** | **Perspective adopted in information** | **Response options** | **Classification of response options** | **Main concepts: What is this information about?** | **Additional concepts contained in the information** | **ICF category of main concept** | **ICF category of other concepts** | **Annotation** |
| --- | --- | --- | --- | --- | --- | --- | --- | --- | --- |
| **PHYSICAL SYMPTOMS** | | | | | | | | | |
| **Chronic dizziness** | "It feels like I am walking on clouds." [UVH-1]  "It's like a momentary slow-motion film strip, like I'm moving slowly." [UVH-2]  "I experience brief dizzy, but it also feels like I am groggy." [UVH-7]  "Does it feel like the environment is spinning? Or is it something else? I feel weak." [UVH-9]  "It feels like one of my feet is in the air, not touching the ground, and when it does touch, it feels like I'm about to fly away." [UVH-14] | Descriptive- Performance | -- | Qualitative attributes | Perception of walking on an unstable surface  Grogginess  Perception of foot not touching the ground  Sensation of swaying  Perception of moving in slow motion | Consciousness  Body position  Proprioception  Energy | **b2351**-Vestibular function of balance  **b2401**-Dizziness | **b110-**Consciousness functions  **b260**-Proprioceptive function  **b130**-Energy and drive functions  **d469**-Walking and moving other specified and unspecified.  **d415**-Maintaning body position  **b156**-Perceptual functions |  |
| **Unsteadiness** | “When I go on bumpy roads, I feel a sense of imbalance, and it feels like I might turn over at any moment." [UVH-2]  "I constantly felt like I was going to fall. I couldn't stay balanced." [UVH-4]  "It always feels like I am leaning to one side. When I was receiving treatment, I tried walking on a straight line, but I would slide to one side." [UVH-10]  "The worst thing I experience is occasional imbalance. I especially feel it more when I am very tired or have not had enough sleep." [UVH-13] | Descriptive- Performance | -- | Qualitative attributes | Unsteadiness on uneven surfaces  Persistent feeling of falling  Loss of balance while walking  Leaning to one side | Fatigue  Lack of sleep | **b2351-**Vestibular function of balance  **b2402**-Sensation of falling  **b2409**-Sensations associated with hearing and vestibular functions, unspecified | **b130**-Energy and drive functions  **b134**-Sleep functions  **d410**-Changing basic body positions  **d460**-Moving around in different locations  **d415**-Maintaining body position  **e298**-Natural environment and human-made changes to environment | Chronic dizziness is more about a subjective feeling of altered spatial orientation, while imbalance is a physical difficulty in maintaining stability. |
| **Supermarket effect** | "For example, colorful objects. Fluid-like things. For instance, black and white things, sliding things." [UVH-2]  "While using my phone, sometimes when scrolling, I get tired and can't follow. It feels like things are coming towards me." [UVH-4]  "When I look at colorful products on the shelves, my balance gets disrupted." [UVH-7]  "When I stir the soup, my eyes spin as the soup spins. My head spins too." [UVH-12] | Descriptive;  Performance | -- | Qualitative attributes | Sensitivity to visual stimuli  Difficulty following moving objects on phone  Sensitivity to fluid-like moving objects  Balance disruption by colorful objects |  | **b210-**Seeing functions  **b2351-**Vestibular function of balance  **b2409**-Sensations associated with hearing and vestibular functions, unspecified  **b2402**-Sensation of falling | **e240-**Light  **b260**-Proprioceptive function  **d110**-Watching  **d160**-Focusing attention  **e120**-Products and technology for personal indoor and outdoor mobility and transportation. | This is called visually-induced dizziness. So, it is unspecified. |
| **Head movements worsen symptoms** | "When I turn quickly my head, I experience sense of losing balance." [UVH-2]  "When I bend over or change my position, for instance, if I bend over to do something and then stand up, if I'm not careful, I might fall." [UVH-5]  "I try to be careful with sudden head movements because I'm afraid I might lose my balance suddenly." [UVH-8]  "I can't look up. Or I try to turn my whole body to look right or left. I can't just turn my neck. Even if I do, I can't see clearly." [UVH-14]  "When I enter a curve quickly, with a quick head movement, my dizziness starts." [UVH-15] | Descriptive;  Performance | -- | Qualitative attributes | Loss of balance with quick movements  Discomfort with head tilting  Restriction in head movement | Precautionary behavior, anxiety  Fear of falling | **b2351**-Vestibular function of balance  **b2409**-Sensations associated with hearing and vestibular functions, unspecified  **d410**-Changing basic body positions  **d110**-Watching  **b760**-Control of voluntary movement functions | **b152**-Emotional functions  **b2402**-Sensation of falling  **d4105-**Bending  **d450**-Walking  **b210-**Seeing functions  **d160**-Focusing attention | This type of symptom is related to dizziness and unsteadiness. However, this is also different entity that specifically the head movements worsen the balance. |
| **Darkness worsens symptoms** | "I struggle to walk in the dark. I think to myself, 'Let me turn on the light ahead before I go,' otherwise I can't find my way in the darkness." [UVH-4]  "I absolutely have to turn on a flashlight or use my phone in the dark. I'm not like I used to be. I have to turn on a light or keep one on in the corridor." [UVH-14] | Descriptive;  Performance | -- | Qualitative attributes | Difficulty walking in the dark | Dependence on external light sources | **d450**- Walking  **b210**- Seeing function  **d460**-Moving around in different locations  **b2351**-Vestibular function of balance  **b21020**-Light sensitivity | **e240**- Light  **d220**- Undertaking multiple tasks |  |
| **Autonomic complaints** | "When I get up suddenly, I experience a loss of balance. To manage this, I try to slow down and control myself." [UVH-12]  "When I stand up quickly, it feels like my blood pressure rises or drops. There’s this sudden pressure in my head. I don't know if it's going up or down, but something happens. Then it passes." [UVH-15] | Descriptive;  Performance | -- | Qualitative attributes | Loss of balance when standing up quickly | Perception of head pressure | **b2351**- Vestibular function of balance  **d410**- Changing basic body position  **b420**-Blood pressure functions |  | The sensation of pressure around neck or head does not take place in ICF. Therefore, it is called not covered (Nc). But it is related to hearing and vestibular functions which is not specified. |
| **Tiredness** | "Sometimes, even if I'm not doing any activity or exerting myself, I find myself feeling tired. I don't know how to prevent this." [UVH-4]  "But I get tired very quickly. I wasn't like this before. I used to go up and down without getting tired. But especially after this dizziness started, it feels like my fatigue has increased." [UVH-6] | Descriptive;  Performance | -- | Qualitative attributes | Fatigue without exertion | Increased fatigue, particularly after dizziness onset | **b4552**- Fatiguability  **b1300**- Energy level | **b2401-** Dizziness |  |
| **Oscillopsia** | "For example, when I'm moving or walking, the objects around me seem to be shaking.” [UVH-2]  "When I'm walking, I notice it, and oddly enough, I kind of enjoy it. Sometimes I even try to enjoy it when I move my head. But it really happens; the things around me seem to be shaking." [UVH-14] | Descriptive;  Performance | -- | Qualitative attributes | Perception of objects shaking while moving |  | **b2100**Visual acuity functions  **d450**-Walking  **b235**- Vestibular functions | **b2401**-Dizziness |  |
| **Tinnitus** | "In the morning, I wake up with a ringing in my ears." [UVH-2]  "My right ear constantly rings, and it's always there." [UVH-5] | Descriptive;  Performance | -- | Qualitative attributes | Sensation of ringing in the ear. |  | **b2400**Ringing in ears or tinnitus |  |  |
| **Recurrent vertigo** | "After my first episode, I didn't have another attack for three years. Then, three years later, I had another one." [UVH-12]  "It always comes in episodes like this. The first one happened seven years ago." [UVH-13] | Descriptive;  Performance | -- | Qualitative attributes | Vertigo attacks occur more than once |  | **b240**Sensations associated with hearing and vestibular functions |  |  |
| **Headache** | "About a week to ten days before the attack, I had a headache where one side was pulling forward, and the other was pulling backward. My head felt like a drum." [UVH-14]  "Recently, in the last few years, I've been getting headaches. For example, I had one this winter." [UVH-15] | Descriptive;  Performance | -- | Qualitative attributes | The sensation of headache |  | **b280**-Sensation of pain |  |  |
| **Hearing loss** | "Now, if I want to listen, I turn my hearing side towards the sound. Otherwise, if someone comes and talks on my other side, I don't hear them at all." [UVH-9]  "I realized later that my right ear wasn't hearing." [UVH-12] | Descriptive;  Performance | -- | Qualitative attributes | Hearing loss  Hearing fluctuation |  | **b230**- Hearing functions |  |  |
| **Brain fog** | "One of my biggest symptoms is when a sudden heaviness and drowsiness comes over me. If I don't sleep at that moment, I know an attack is coming. I start feeling like I'm in a daze." [UVH-2] "It's just like I suddenly feel dazed, like I'm in a fog." [UVH-6] | Descriptive;  Performance | -- | Qualitative attributes | Lack of mental clarity and a feeling of being mentally disoriented. |  | **b144**-Memory functions |  |  |
| **Neck pain** | "Especially after that period, about 1 or 2 months later, a dull pain remained at the back of my head. It's like the kind of stiffness you feel in some organs." [UVH-3] | Descriptive;  Performance | -- | Qualitative attributes | Neck pain |  | **b28010**Pain in head and neck |  |  |
| **Sweating** | "Even if I don't move much, I sweat. My sweating has become more frequent." [UVH-5] | Descriptive;  Performance | -- | Qualitative attributes |  |  | **b830**- Other functions of the skin |  | This category includes the functions of sweating in ICF. |
| **Aural fullness** | "But I also feel like there's a sense of fullness in my right ear." [UVH-6] | Descriptive;  Performance | -- | Qualitative attributes |  |  | **b2405**- Aural pressure |  |  |
| **COGNITIVE SYMPTOMS** | | | | | | | | | |
| **Difficulties with dual tasks** | "I usually focus on one task first, finish it, and then move on to the next activity. It takes more time this way, but if I try to do both at the same time, everything ends up a mess." [UVH-4]  "For example, when my wife is talking to me, I can't do something else at the same time. I need to focus on listening to her." [UVH-5]  “I can't exactly figure out where to focus when I have two tasks at once. For example, I can't concentrate on my phone while I'm walking” [UVH-8] | Descriptive;  Performance | -- | Qualitative attributes | Difficulty with multitasking  Distraction  Lack of focus |  | **b140**Attention functions  **d220**-Undertaking multiple tasks | **d310** - Communicating with - receiving - spoken messages  **d450** - Walking |  |
| **Disorientation** | "I often find myself getting confused about directions, like not knowing which way to turn in familiar places." [UVH-5] | Descriptive;  Performance | -- | Qualitative attributes | Confusion  Navigation issues |  | **b114**-Orientation functions |  |  |
| **Misjudging distances** | "When I'm driving—I try to line up the car while overtaking, but I still end up touching the car" [UVH-5]  "I've had a few issues like occasionally bumping into the elevator door while walking or hitting my arm while passing through a doorway." [UVH-7] | Descriptive;  Performance | -- | Qualitative attributes | Difficulty with driving |  | **b164**-Higher-level cognitive functions |  |  |
| **Concentration problems** | "I can't keep up with long conversations after a while. They start to bore and tire me out." [UVH-8]  "Sometimes, while reading, I find myself not understanding, like my mind is elsewhere. I often have to read things twice or struggle to fully concentrate" [UVH-10]  "Even with something I'm familiar with, I often find myself having to repeat the same task or process." [UVH-14] | Descriptive;  Performance | -- | Qualitative attributes | Fatigue  Disinterest  Repetition |  | **b140**-Attention functions  **b144-**Memory functions  **b160-**Thought functions | **d350-** Conversation  **d166-**Reading |  |
| **Forgetfulness** | "My memory used to be excellent; I never forgot a face or a voice. Now, I forget things easily and often have to repeat what I read to understand it." [UVH-2]  "While praying, I used to never forget the verses, but in the past couple of years, I've found myself mixing up which ones I've recited or which part of the prayer I'm on." [UVH-5]  "Sometimes I forget tasks I need to do during the day, even when I'm not busy. When I remember later, I get frustrated with myself for forgetting." [UVH-9] | Descriptive;  Performance | -- | Qualitative attributes | Repetition  Memory lapses  Confusion |  | **b144-**Memory functions | **d230-**Carrying out daily routine |  |
| **EMOTIONS** | | | | | | | | | |
| **Disappointment** | "I didn't expect to have this illness" [UVH-6] | Descriptive;  Performance | -- | Qualitative attributes | Disappointment |  | **b152 -** Emotional functions |  |  |
| **Displeasure** | "My wife never leaves me alone due to my balance issues; it makes me feel dependent" [UVH-4] | Descriptive;  Performance | -- | Qualitative attributes | Feeling dependent |  | **b152 -** Emotional functions | **d760** - Family relationships |  |
| **Embarrasment** | "I feel concerned if others notice when I stumble" [UVH-3] | Descriptive;  Performance | -- | Qualitative attributes | Concern about judgment |  | **b152 -** Emotional functions  **e460**- Societal attitudes | **d730** Relating with strangers |  |
| **Insecurity** | "I feel unsure of myself, what if something happens spontaneously?" [UVH-2] | Descriptive;  Performance | -- | Qualitative attributes | Insecurity |  | **b152 -** Emotional functions  **b126**-Temperament and personality functions |  |  |
| **Isolation** | "This condition makes me feel trapped" [UVH-7] | Descriptive;  Performance | -- | Qualitative attributes | Feeling trapped |  | **b152 -** Emotional functions  **d240**-Handling stress and other psychological demands |  |  |
| **Sadness** | "Family issues make me sad" [UVH-12] | Descriptive;  Performance | -- | Qualitative attributes | Sadness |  | **b152 -** Emotional functions | **d760** - Family relationships |  |
| **Depression** | "A sudden heaviness and drowsiness overwhelms me" [UVH-2] | Descriptive;  Performance | -- | Qualitative attributes | Depression |  | **b152 -** Emotional functions |  |  |
| **Despair** | "I asked the doctor if I could drive a car again" [UVH-11] | Descriptive;  Performance | -- | Qualitative attributes | Despair |  | **b152 -** Emotional functions | **d475** - Driving |  |
| **Woe** | "Constant dizziness makes me feel I've lost control" [UVH-8] | Descriptive;  Performance | -- | Qualitative attributes | Feeling of loss of control |  | **b152 -** Emotional functions | **b235**-Vestibular functions |  |
| **Suffering** | "It feels like my mind is constantly heavy, and I can't find any relief." [UVH-10] | Descriptive;  Performance | -- | Qualitative attributes | Mental suffering |  | **b152 -** Emotional functions  **b130**-Energy and drive functions |  |  |
| **Anxiety** | "I'm afraid of going out alone, anxious about facing the same challenges" [UVH-1] | Descriptive;  Performance | -- | Qualitative attributes | Anxiety |  | **b152 -** Emotional functions | **d460** - Moving around in different locations |  |
| **Distress** | "I have to focus on my condition because my quality of life deteriorates" [UVH-2] | Descriptive;  Performance | -- | Qualitative attributes | Distress |  | **b152 -** Emotional functions | **b160** - Thought functions |  |
| **Worry** | "I'm worried about falling or hurting myself" [UVH-7] | Descriptive;  Performance | -- | Qualitative attributes | Worry |  | **b152 -** Emotional functions | **b2402** Sensation of falling |  |
| **Fear** | "I try to avoid darkness; it terrifies me" [UVH-5] | Descriptive;  Performance | -- | Qualitative attributes | Fear of darkness |  | **b152 -** Emotional functions | **e240** - Light |  |
| **Panic** | "Sometimes, I suddenly feel overwhelmed, like my heart is racing and I can't catch my breath." [UVH-2]. | Descriptive;  Performance | -- | Qualitative attributes | Overwhelming fear |  | **b152 -** Emotional functions | **b420** Blood pressure functions |  |
| **Frustration** | "My tolerance level has decreased, and I react strongly to situations" [UVH-4]. | Descriptive;  Performance | -- | Qualitative attributes | Frustration |  | **b152 -** Emotional functions |  |  |
| **Irritation** | "Having my movements restricted irritates me" [UVH-10] | Descriptive;  Performance | -- | Qualitative attributes | Irritation |  | **b152 -** Emotional functions | **d415**-Maintaning a body position |  |
| **Happiness** | "At least I'm happy that this disease is gradually getting better." [UVH-8] | Descriptive;  Performance | -- | Qualitative attributes | Happiness |  | **b152 -** Emotional functions |  |  |
| **Contentment** | "I’ve learned to appreciate life and be content with what I have" [UVH-9] | Descriptive;  Performance | -- | Qualitative attributes | Contentment |  | **b152 -** Emotional functions |  |  |
| **Optimism** | "I comfort myself by believing this illness will eventually improve" [UVH-7] | Descriptive;  Performance | -- | Qualitative attributes | Optimism |  | **b152 -** Emotional functions |  |  |
| **Fondness** | "I care for my family; their company brings me joy" [UVH-4] | Descriptive;  Performance | -- | Qualitative attributes | Fondness |  | **b152 -** Emotional functions | **d760** - Family relationships |  |
| **Sentimentality** | "Support from my close family keeps me going" [UVH-6] | Descriptive;  Performance | -- | Qualitative attributes | Sentimentality |  | **b152 -** Emotional functions | **d760** - Family relationships  **e310**-Immediate family |  |
| **CHALLENGING TASKS** | | | | | | | | | |
| **Driving** | "I get dizzy when I turn my head suddenly while driving." [UVH-7]  "Sometimes it feels like my vision becomes distorted or unusual when I'm driving a car." [UVH-12]  "I have trouble with sharp turns while driving; they seem to trigger me." [UVH-15] | Descriptive;  Performance | -- | Qualitative attributes | Difficulty when turning head  Visual vertigo  Worsening symptoms on uneven ground, sharp turns |  | **d475**- Driving  **b210-** Seeing function  **b2401-** Dizziness |  |  |
| **Sleeping problem** | "Sometimes I wake up very often during the night. Sometimes I can sleep comfortably until the morning. As I said, there is a lack of consistency." [UVH-9]  "I have a very hard time falling asleep, unfortunately. It's very interrupted, I jump at even the slightest sound. I can't remember a time when I had a deep, uninterrupted sleep." [UVH-11] | Descriptive;  Performance | -- | Qualitative attributes | \| Difficulty maintaining consistent sleep  Difficulty falling asleep \| \| --- \|  \|  \| \| --- \| |  | **b1341-** Onset of sleep  **b1342-** Maintenance of sleep |  |  |
| **Fear of falling** | "When there are two tasks at the same time, I can't fully decide what to focus on. I have fears that I might lose my balance." [UVH-8]‬  "I already don't like the dark at all. And when I get up in the dark, I have this fear of falling." [UVH-14]‬  "I can go down, but probably a bit slowly. I can go down, but I might be scared. I can go up without holding on, but when going down, it might feel like I'm about to fall." [UVH-15]‬ | Descriptive;  Performance | -- | Qualitative attributes | Dual-task difficulty  Fear of falling  Needing light | Dual-task  Light  Scared | **b140** Attention functions  **b2402** Sensation of falling  **b235**-Vestibular functions | **e240-** Light  **d410-** Changing basic body positions  **b152**-Emotinal functions  **d220**-Undertaking multiple tasks |  |
| **Disturbance in crowded environments** | "We are in a crowded group setting, you are talking, the other person is talking, and another person is talking. That triggers an attack for me." [UVH-2]  "I can enter the supermarket comfortably, but when there are a lot of people, that disturbs me a little." [UVH-8]  "Let me put it this way, sounds that I don't like bother me. For example, the constant honking in traffic, or even music I don't want to hear can be disturbing." [UVH-9]‬  "Because of my ear, since I have trouble hearing, noise also affects me." [UVH-12]‬ | Descriptive;  Performance | -- | Qualitative attributes | Discomfort in crowded places  Hearing difficulties and sensitivity to noise | Sound  Disturbing | **b230** Hearing functions  **b240** Sensations associated with hearing and vestibular functions  **d750**- Informal social relationships | **e250**-Sound  **b152**-Emotional functions |  |
| **Reading difficulties** | "When I'm watching TV at home, I especially have a hard time reading the subtitles when they appear." [UVH-2]‬ | Descriptive;  Performance | -- | Qualitative attributes | Difficulty reading subtitles on TV |  | **d166-**Reading  **b210-**Seeing function | **d110-**Watching  **e125**-Products and technology |  |
| **BEHAVIORS** | | | | | | | | | |
| **Acceptance of condition** | "I accept that as I get older, there will be some difficulties in life. After all, I've reached a certain age." [UVH-8]  "I think a lot about the ear issue myself. I handled it very calmly. I mean, all the bad things that happened, the surgeries, the deafness, I accepted it. I have learned to live with it." [UVH-11]  "Well, maybe it used to happen in the beginning, but now because I’ve accepted it. I've accepted myself this way. Therefore, I know what I need to do for myself, or what I shouldn't do." [UVH-15] | Descriptive;  Performance | -- | Qualitative attributes | Acceptance of condition (related to ear issue)  Demonstration of emotion related to condition |  | **b126-**Temperament and personality functions  **b152-**Emotional functions | **d240**-Handling stress and other psychological demands |  |
| **Less interaction** | "Even the time I spend with my child has changed" [UVH-2]. | Descriptive;  Performance | -- | Qualitative attributes | Reduced interaction with family |  | **d760-**Family relationships |  |  |
| **Limitations in daily life activities** | "If there's something tiring, I don't enter that environment. If there's an event happening or tasks to be done together, I'm involved in the least part of it." [UVH-2]  "I don't have my old activities and movements anymore. I used to work a lot, but I can't work the way I used to." [UVH-5]  "After this illness, whether it's traveling, my work environment, or evening dinners... Of course, it has been a hindrance. Suddenly, I felt like I was in a void. Like, as they say, I became like someone in prison." [UVH-7]  "I used to meet with my neighbors more often, but this condition has affected me." [UVH-12] | Descriptive;  Performance | -- | Qualitative attributes | Avoidance of overwhelming activities  Reduced capacity for work and physical activities  Reduced social interactions due to health condition |  | **d910-**Community life  **b130-**Energy and drive functions  **d920-**Recreation and leisure  **b152-**Emotional functions  **d750-**Informal social relationships  **d760-**Family relationships | **d850-**Remunerative employment |  |
| **COPING STRATEGIES** | | | | | | | | | |
| **Coping strategies** | "I now think that I will do everything without rushing, taking slow steps." [UVH-2]  "I try to protect myself as much as possible, avoiding rushing, overexertion, and noisy environments." [UVH-2]  "When I stand up, I wait a few seconds before walking. I hold the railing when using stairs, watch my diet, go for walks, and swim." [UVH-4]  "When praying, I keep a chair nearby for support when standing up." [UVH-5]  "I’ve developed strategies like moving slowly, getting up carefully, and taking my time." [UVH-7]  "When going up and down stairs, I make sure to hold onto something for support." [UVH-7]  "I remind myself to walk cautiously after getting up, and to sit for a moment before moving." [UVH-9]  "At night, I support myself by holding onto walls when moving around." [UVH-10]  "I completely avoid sudden movements and always hold onto something when using stairs." [UVH-11]  "If I go out for a long time, I try not to go alone and avoid standing too long." [UVH-1]  "I always hold onto something when going up or down stairs." [UVH-12]  "When I see a lot of movement, I lose balance. I sit down and focus on a single point until I feel stable." [UVH-13]  "I never lie on my left side, and when I need to look to the left, I turn my whole body. I avoid looking up. I try to do tasks like changing light bulbs without raising my head." [UVH-15] | Descriptive;  Performance | -- | Qualitative attributes | Moving slowly to prevent symptoms  Self-protection  Cautious movement and support use when standing or using stairs  Using walls for support at night | Prevention of falls on stairs  Avoiding prolonged standing alone  Noisy environment | **b760-**Control of voluntary movement functions  **d410-**Changing body position  **d460-**Moving around in different locations  **b2351-**Vestibular functions of balance  **d160-**Focusing attention | **e298-**Natural environment and human-made changes to environment  **e150-**Design, construction, and building products and technology of buildings for public use  **e115**-Products and technology for personal use in daily living |  |

**Table S4.** The extracted data and linking details from PROMs (DHI, HADS and EQ-5D-5L)

| **Name of symptoms** | **Verbatim health information** | **Perspective adopted in information** | **Response options** | **Classification of response options** | | **Main concepts: What is this information about?** | **Additional concepts contained in the information** | **ICF category of main concept** | **ICF category of other concepts** | **Annotation** |
| --- | --- | --- | --- | --- | --- | --- | --- | --- | --- | --- |
| **Dizziness Handicap Inventory (DHI)** | | | | | | | | | | |
| **DHI** | 1-Does looking up increase your problem? | Descriptive- Capacity | Yes, Sometimes, No | Intensity | | Looking up  Change head position | Dizziness | **d110-** Watching **d410-** Changing body positions | **b2401-** Dizziness | Problem means already dizziness |
| **DHI** | 2- Because of your problem, do you feel frustrated? | Appraisal | Yes, Sometimes, No | Intensity | | Feeling frustrated | Dizziness | **b152**- Emotional functions | **b2401**- Dizziness |  |
| **DHI** | 3-Because of your problem, do you restrict your travel for business or recreation? | Descriptive- Capacity | Yes, Sometimes, No | Intensity | | Restriction in travel | Dizziness | **d470**-Using transportation **d475**- Driving | **b2401**- Dizziness |  |
| **DHI** | 4-Does walking down the aisle of a supermarket increase your problems? | Descriptive- Performance | Yes, Sometimes, No | Intensity | | Walking in a supermarket | Dizziness | **d4601**- Moving around within buildings other than home | **e150**- Design, construction and building products and technology of buildings for public use b2401- Dizziness |  |
| **DHI** | 5-Because of your problem, do you have difficulty getting into or out of bed? | Descriptive- Capacity | Yes, Sometimes, No | Intensity | | Difficulty getting into or out of bed | Dizziness | **d410**-Changing body position | **b2401**- Dizziness |  |
| **DHI** | 6-Does your problem significantly restrict your participation in social activities, such as going out to dinner, going to the movies, dancing, or going to parties? | Descriptive- Performance | Yes, Sometimes, No | Intensity | | Restriction in social participation | Dizziness | **d920**- Recreation and leisure | **b2401**- Dizziness |  |
| **DHI** | 7-Because of your problem, do you have difficulty reading? | Descriptive- Capacity | Yes, Sometimes, No | Intensity | | Difficulty reading | Dizziness | **d166**- Reading | **b2401**- Dizziness |  |
| **DHI** | 8-Does performing more ambitious activities such as sports, dancing, household chores (sweeping or putting dishes away) increase your problems? | Descriptive- Performance | Yes, Sometimes, No | Intensity | | Difficulty with ambitious activities | Dizziness | **d9201**- Sports **d640**- Doing housework | **b7602**- Coordination of voluntary movement **b730**- Muscle power functions **b2401**- Dizziness |  |
| **DHI** | 9-Because of your problem, are you afraid to leave your home without having someone accompany you? | Descriptive- Capacity | Yes, Sometimes, No | Intensity | | Fear of leaving home alone | Dizziness | **b152**- Emotional functions **e340**- Personal care providers and personal assistans | **b2401**- Dizziness |  |
| **DHI** | 10-Because of your problem have you been embarrassed in front of others? | Appraisal | Yes, Sometimes, No | Intensity | | Embarrassment | Dizziness | **b152**- Emotional functions **e460**- Societal attitudes | **b2401**- Dizziness |  |
| **DHI** | 11-Do quick movements of your head increase your problem? | Descriptive-Capacity | Yes, Sometimes, No | Intensity | | Quick head movements | Dizziness | **b760**- Control of voluntary movement functions | **b2401**- Dizziness |  |
| **DHI** | 12-Because of your problem, do you avoid heights? | Descriptive- Performance | Yes, Sometimes, No | Intensity | | Avoidance of heights | Dizziness | **e2100**-Land forms | **e150**- Design, construction and building products and technology of buildings for public use b2401- Dizziness |  |
| **DHI** | 13-Does turning over in bed increase your problem? | Descriptive-Capacity | Yes, Sometimes, No | Intensity | | Turning over in bed | Dizziness | **d410**- Changing body position | **b2401**- Dizziness |  |
| **DHI** | 14-Because of your problem, is it difficult for you to do strenuous homework or yard work? | Descriptive- Performance | Yes, Sometimes, No | Intensity | | Difficulty with strenuous work | Dizziness | **d640**- Doing houseworks | **b730**- Muscle power functions **b740**- Muscle endurance functions **b2401**- Dizziness |  |
| **DHI** | 15- Because of your problem, are you afraid people may think you are intoxicated? | Descriptive-Capacity | Yes, Sometimes, No | Intensity | | Fear of being judged | Dizziness | **b152**- Emotional functions **e460**- Societal attitudes | **b2401**- Dizziness |  |
| **DHI** | 16-Because of your problem, is it difficult for you to go for a walk by yourself? | Appraisal | Yes, Sometimes, No | Intensity | | Difficulty walking alone | Dizziness | **d4602**- Moving around outside the home and other buildings **e340**- Personal assistants | **b2401**- Dizziness |  |
| **DHI** | 17-Does walking down a sidewalk increase your problem? | Descriptive-Capacity | Yes, Sometimes, No | Intensity | | Walking on a sidewalk | Dizziness | **d4602**- Moving around outside the home and other buildings | **e298**- Natural environment and human-made changes to environment **b2401**- Dizziness |  |
| **DHI** | 18-Because of your problem, is it difficult for you to concentrate? | Descriptive- Performance | Yes, Sometimes, No | Intensity | | Difficulty concentrating | Dizziness | **d160**- Focusing attention | **b2401**- Dizziness |  |
| **DHI** | 19-Because of your problem, is it difficult for you to walk around your house in the dark? | Descriptive-Capacity | Yes, Sometimes, No | Intensity | | Walking in the dark | Dizziness | **d4600**- Moving around within the home | **e240**- Light **b2401**- Dizziness |  |
| **DHI** | 20-Because of your problem, are you afraid to stay home alone? | Descriptive- Performance | Yes, Sometimes, No | Intensity | | Fear of staying home alone | Dizziness | **b152**- Emotional functions **e340**- Personal care providers and personal assistans | **b2401**- Dizziness |  |
| **DHI** | 21-Because of your problem, do you feel handicapped? | Descriptive-Capacity | Yes, Sometimes, No | Intensity | | Feeling handicapped | Dizziness | **b11420**- Orientation to self **b180**- Experience of self and time functions | **b2401**- Dizziness |  |
| **DHI** | 22-Has the problem placed stress on your relationships with members of your family or friends? | Appraisal | Yes, Sometimes, No | Intensity | | Stress in relationships | Dizziness | **d750**- Informal social relationship **d760**- Family relationship | **b2401**- Dizziness |  |
| **DHI** | 23-Because of your problem, are you depressed? | Descriptive-Capacity | Yes, Sometimes, No | Intensity | | Feeling depressed | Dizziness | **b152**- Emotional functions | **b2401**- Dizziness |  |
| **DHI** | 24-Does your problem interfere with your job or household responsibilities? | Descriptive- Performance | Yes, Sometimes, No | Intensity | | Interference with responsibilities | Dizziness | **d640**- Doing houseworks **d850**- Remunerative employment | **b2401**- Dizziness |  |
| **DHI** | 25- Does bending over increase your problem? | Descriptive-Capacity | Yes, Sometimes, No | Intensity | | Bending over | Dizziness | **d4105**- Bending | **b2401**- Dizziness |  |
| **Hospital Anxiety and Depression Scale (HADS)** | | | | | | | | | | |
| **HADS** | 1-I feel tense or 'wound up' | Appraisal | Most of the time, A lot of the time, From time to time, occasionally, Not at all | | Intensity | Feel tense |  | **b152** - Emotional functions |  |  |
| **HADS** | 2-I still enjoy the things I used to enjoy | Appraisal | Definitely as much, Not quite so much, Only a little, Hardly at all | | Intensity | Enjoy |  | **b152** - Emotional functions |  |  |
| **HADS** | 3-I get a sort of frightened feeling as if something awful is about to happen | Appraisal | Very definitely and quite badly, Yes but not too badly, A little but it doesn't worry me, Not at all | | Intensity | Feeling frightened |  | **b152** - Emotional functions |  |  |
| **HADS** | 4- I can laugh and see the funny side of things | Appraisal | As much as I always could, Not quite so much now, Definitely not so much now, Not at all | | Intensity | Laughing |  | **b152** - Emotional functions |  |  |
| **HADS** | 5-Worrying thoughts go through my mind: | Appraisal | A great deal of the time, A lot of the time, From time to time but not too often, Only occasionally | | Intensity | Worrying thoughts |  | **b152** - Emotional functions | **b140**-Attention functions |  |
| **HADS** | 6-I feel cheerful | Appraisal | Not at all, Not often, Sometimes, Most of the time | | Intensity | Feeling cheerful |  | **b152** - Emotional functions |  |  |
| **HADS** | 7-I can sit at ease and feel relaxed | Appraisal | Definitely, Usually, Not often, Not at all | | Intensity | Feeling relaxed |  | **b152** - Emotional functions |  |  |
| **HADS** | 8-I feel as if I am slowed down | Appraisal | Nearly all the time, Very often, Sometimes, Not at all | | Intensity | Feeling slowed down |  | **b152** - Emotional functions | **b130**-Energy and drive functions |  |
| **HADS** | 9- I get a sort of frightened feeling like 'butterflies' in the stomach | Appraisal | Not at all, Occasionally, Quite often, Very often | | Intensity | Feeling frightened |  | **b152** - Emotional functions |  |  |
| **HADS** | 10-I have lost interest in my appearance | Appraisal | Definitely, I don't take as much care as I should, I may not take quite as much care, I take just as much care as ever | | Intensity | Loss of interest in appearance |  | **b152** - Emotional functions |  |  |
| **HADS** | 11-I feel restless as I have to be on the move | Appraisal | Very much indeed, Quite a lot, Not very much, Not at all | | Intensity | Feeling restless |  | **b152** - Emotional functions |  |  |
| **HADS** | 12-I look forward with enjoyment to things | Appraisal | As much as I ever did, Rather less than I used to, Definitely less than I used to, Hardly at all | | Intensity | Anticipation of enjoyment |  | **b152** - Emotional functions | **d920** - Recreation and leisure |  |
| **HADS** | 13-I get sudden feelings of panic | Appraisal | Very often indeed, Quite often, Not very often, Not at all | | Intensity | Feeling panic |  | **b152** - Emotional functions |  |  |
| **HADS** | 14-I can enjoy a good book or radio or TV program | Appraisal | Often, Sometimes, Not often, Very seldom | | Intensity | Enjoyment of leisure activities |  | **b152** - Emotional functions | **d920** - Recreation and leisure |  |
| **EuroQoL- 5 Dimension- 5 Level (EQ-5D-5L)** | | | | | | | | | | |
| **EQ-5D-5L** | 1-Mobility | Descriptive- Capacity | 1,2,3,4,5 | Intensity | | Walking |  | **d450** - Walking |  |  |
| **EQ-5D-5L** | 2-Self-care | Descriptive- Capacity | 1,2,3,4,5 | Intensity | | Washing  Dressing |  | **d510**- Washing oneself  **d540**- Dressing |  |  |
| **EQ-5D-5L** | 3-Usual activities | Descriptive- Performance | 1,2,3,4,5 | Intensity | | Usual activities | Working Studying Housework Family Leisure activities | **d230**- Carrying out daily routine | **d720**- Complex interpersonal interactions **d760**- Family relationships **d640**-Doing housework **d920**- Recreation and leisure | Working and studying  are considered under  the complex interpersonal  interactions |
| **EQ-5D-5L** | 4-Pain/Discomfort | Appraisal | 1,2,3,4,5 | Intensity | | Pain or discomfort |  | **b280**- Sensation of pain | **d760** - Family relationships  **e310**-Immediate family |  |
| **EQ-5D-5L** | 5-Anxiety/Depression | Appraisal | 1,2,3,4,5 | Intensity | | Anxiety or depression |  | **b1529**- Emotional functions, unspecified |  |  |

**Table S5.** Chronic unilateral vestibular hypofunction symptoms: Physical, cognitive and emotions

| **Semptoms** | **Percentage (%)** | **N** |  |
| --- | --- | --- | --- |
| **Physical**   - Chronic dizziness - Imbalance (Unsteadiness) - Supermarket effect - Head movements worsen symptoms - Recurrent vertigo - Tinnitus - Darkness worsens symptoms - Autonomic complaints - Tiredness - Headache - Hearing loss - Brain fog - Neck pain - Sweating - Aural fulness - Oscillopsia | 80%  80%  73%  73%  53%  47%  40%  40%  33%  33%  33%  27%  20%  20%  13%  13% | 12/15  12/15  11/15  11/15  8/15  7/15  6/15  6/15  5/15  5/15  5/15  4/15  3/15  3/15  2/15  2/15 |  |
| **Cognitive**   - Concentration problems - Forgetfulness - Difficulties with dual tasking - Problems with spatial orientation   - - Disorientation     - Misjudging distances | 67%  40%  27%  13%  13% | 10/15  6/15  4/15  2/15  2/15 |  |
| **Emotions** |  |  |  |
| - **Sadness**   - Suffering     - Suffering   - Sadness     - Sadness     - Depression     - Despair     - Woe   - Neglect     - Embarrasment     - Isolation     - Insecurity   - Disappointment     - Disappointment     - Displeasure | **73%**  7%  47%  20%  60% | **11/15**  1/15  7/15  2/15  9/15 |  |
| - **Fear**   - Horror     - Fear     - Horror     - Panic   - Nervousness     - Anxiety     - Distress     - Worry | **60%**  13%  60% | **9/15**  2/15  9/15 | |
| - **Anger**   - Exasperation     - Exasperation     - Frustration   - Irritation     - Irritation     - Annoyance | **53%**  47%  20% | **8/15**  7/15  3/15 | |
| - **Joy**   - Cheerfulness     - Happiness   - Contentment     - Contentment   - Optimism     - Optimism     - Hope     - Eagerness | **27%**  7%  13%  13% | **4/15**  1/15  2/15  2/15 | |
| - **Love**   - Affection     - Fondness     - Sentimentality     - Affection | **20%**  20% | **3/15**  3/15 | |
